# Pulse oximetry values from 33,080 participants in the Apple Heart & Movement Study

**DOI:** 10.1038/s41746-023-00851-6

**Published:** 2023-07-27

**Authors:** Ian Shapiro, Jeff Stein, Calum MacRae, Michael O’Reilly

**Affiliations:** 1 Apple Inc., Cupertino, CA USA; 2 Cardiovascular Medicine Division, Brigham and Women’s Hospital, Boston, MA USA; 3 Harvard Medical School, Boston, MA USA

**Keywords:** Physiology, Cardiovascular biology

## Abstract

Wearable devices that include pulse oximetry (SpO_2_) sensing afford the opportunity to capture oxygen saturation measurements from large cohorts under naturalistic conditions. We report here a cross-sectional analysis of 72 million SpO_2_ values collected from 33,080 individual participants in the Apple Heart and Movement Study, stratified by age, sex, body mass index (BMI), home altitude, and other demographic variables. Measurements aggregated by hour of day into 24-h SpO_2_ profiles exhibit similar circadian patterns for all demographic groups, being approximately sinusoidal with nadir near midnight local time, zenith near noon local time, and mean 0.8% lower saturation during overnight hours. Using SpO_2_ measurements averaged for each subject into mean nocturnal and daytime SpO_2_ values, we employ multivariate ordinary least squares regression to quantify population-level trends according to demographic factors. For the full cohort, regression coefficients obtained from models fit to daytime SpO_2_ are in close quantitative agreement with the corresponding values from published reference models for awake arterial oxygen saturation measured under controlled laboratory conditions. Regression models stratified by sex reveal significantly different age- and BMI-dependent SpO_2_ trends for females compared with males, although constant terms and regression coefficients for altitude do not differ between sexes. Incorporating categorical variables encoding self-reported race/ethnicity into the full-cohort regression models identifies small but statistically significant differences in daytime SpO_2_ (largest coefficient corresponding to 0.13% lower SpO_2_, for Hispanic study participants compared to White participants), but no significant differences between groups for nocturnal SpO_2_. Additional stratified analysis comparing regression models fit independently to subjects in each race/ethnicity group is suggestive of small differences in age- and sex-dependent trends, but indicates no significant difference in constant terms between any race/ethnicity groups for either daytime or nocturnal SpO_2_. The large diverse study population and study design employing automated background SpO_2_ measurements spanning the full 24-h circadian cycle enables the establishment of healthy population reference trends outside of clinical settings.

## Introduction

Arterial blood oxygen saturation (SaO_2_) is the fraction of hemoglobin containing bound oxygen relative to the total functional hemoglobin, and represents a key parameter indicative of cardiopulmonary function. Direct SaO_2_ measurement necessitates an invasive arterial blood draw and blood gas analysis. Pulse oximetry enables non-invasive measurement of blood oxygen saturation (SpO_2_) and provides a convenient estimate of SaO_2_ that does not require arterial blood removal. The SpO_2_ measurement relies upon quantifying changes in optical attenuation at two separate wavelengths (typically one red and one infrared), with signal content arising from pulsatile arterial blood modulation in response to individual heartbeats. Depending on design, pulse oximeters may operate in either transmissive mode, with the interrogating light propagating across a thin section of capillary rich tissue (commonly fingertip, earlobe, or toe), or in reflectance mode wherein the interrogating light scatters back in the direction of the optical illuminator. Reflectance SpO_2_ is employed by consumer smart watch devices such as the Apple Watch (selected models) as well as selected products from Fitbit, Garmin, Samsung, Withings, and other manufacturers.

Oxygen saturation determined from SaO_2_ or SpO_2_ is often considered a ”fifth vital sign” due to its relative ease of capture and high clinical utility^1,2^. As a physiological metric, arterial oxygen saturation directly impacts systemic oxygen delivery in conjunction with cardiac output and hemoglobin concentration. Among healthy awake individuals, typical SpO_2_ values lie in the range of 95–99%. Low blood oxygen saturation can arise from impaired lung function (e.g., reduced diffusion capacity), ventilation-perfusion mismatch, cardiac shunt, low cardiac output, or low oxygen concentration in the inspired air (e.g., due to altitude). No single universal SpO_2_ threshold is applied in all medical use cases, but values less than 92% from individuals breathing room air at sea level generally prompt further investigation, with values remaining persistently below 90% indicating hypoxemia. Oxygen saturation is utilized to guide management of cardiopulmonary conditions such as chronic obstructive pulmonary disease (COPD), obesity hypoventilation syndrome (OHS), and obstructive sleep apnea (OSA).

Cross-sectional studies involving single-setting SpO_2_ or SaO_2_ measurements from nominally healthy individuals at constant altitude have consistently reported negative correlation of blood oxygen saturation with both age and body mass^3–7^. Studies incorporating multiple altitudes or a range of barometric pressure consistently report a positive linear relationship between awake arterial oxygen saturation and barometric pressure, in agreement with expectations based on the alveolar gas equation^3,8,9^. Less consistently, some studies have also reported positive correlation between SpO_2_ and female sex^5,10,11^, although others have reported negative or insignificant SpO_2_ findings with respect to sex^12^. A similar mix of conclusions has been published with respect to tobacco smoking status, with some studies reporting lower SpO_2_ values for current smokers^6^ and others reporting no significant relationship^5^.

In the context of clinical screening and risk estimation for chronic cardiopulmonary disease, single-point SpO_2_ measurements below 95% saturation have been reported as predictive of a variety of cardiopulmonary conditions and outcomes^13–18^. The Tromsø Study examined single-event SpO_2_ values and 10-year outcomes for cardiopulmonary disease, reporting significant elevated risk for values ≤92% and 93–95% saturation, compared with 96–100% saturation^14^. Daytime SpO_2_ has been reported as a significant independent predictor of hypertension^13^, as well as circulatory impairment in the form of impaired left ventricular filling^15^. Mean overnight SpO_2_ has also been reported as predictive of both absolute waking blood pressure and magnitude of morning blood pressure surge^16^. Studies examining overnight SpO_2_ in the context of atherosclerotic cardiovascular risk have produced inconsistent findings, with some reporting significant relationships between mean overnight SpO_2_ and presence of carotid artery plaque^17^, and others reporting no significant relationship after adjusting for demographic variables and other known risk factors^18^.

In the present study we analyze systematic variation in mean daytime and nocturnal SpO_2_ captured by wearable devices, stratified by age, gender, body mass index (BMI), home altitude, and other self-identified demographic factors including race and ethnicity. All subject groups exhibit approximately sinusoidal variation in mean SpO_2_, with highest values in mid-day and mean 0.8% lower saturation during nocturnal hours compared to daytime hours. We employ linear regression models to quantify these trends and enable comparison with existing published reference equations developed from smaller studies utilizing arterial blood gas analysis^3,19^ and pulse oximetry^20^. Both daytime SpO_2_ and nocturnal SpO_2_ exhibit a progressive decline with increasing age, BMI and home altitude. Compared with daytime SpO_2_, nocturnal SpO_2_ regression models yield higher coefficients of determination and emphasize the effects of age, BMI, and altitude in all subject groups. Additionally, the large subject pool in this study enables us to detect small but significant differences in age- and BMI-dependent trends in SpO_2_ between sexes, with female subjects displaying a greater rate of age-dependent decline in both daytime SpO_2_ and nocturnal SpO_2_.

## Results

### Population distributions of mean daytime and nocturnal SpO_2_

**Fig. 1:**
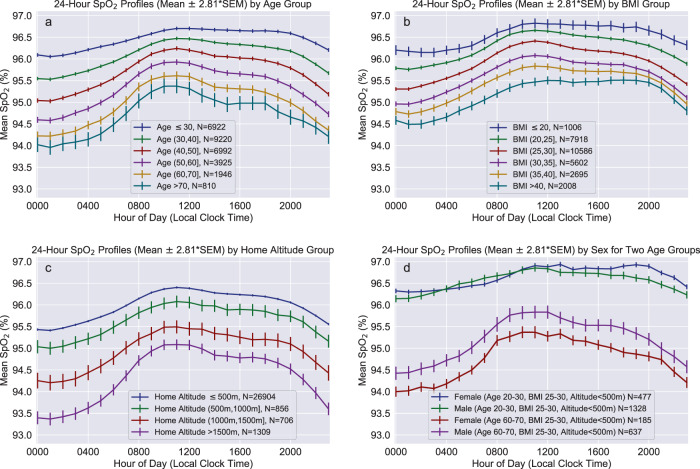
Twenty-four-hour group mean SpO_2_ profiles stratified by demographic variables and home altitude. Profiles are stratified according to **a** age, **b** body mass index, **c** home altitude and **d** assigned sex for two age groups with limited range of BMI and home altitude. Solid lines indicate group mean value for each hour, with whiskers indicating ±2.81 times the SEM (equivalent to 99.5% confidence interval for the mean value) for each hour. Group profiles were determined by first generating the hourly SpO_2_ profile for each subject, then calculating the mean and SEM across subjects for each hour as described in the “Methods” section. SEM standard error of the mean.

**Fig. 2:**
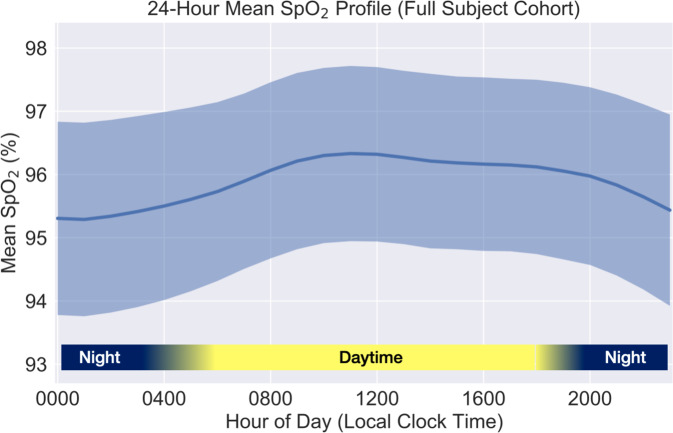
Twenty-four-hour SpO_2_ variation for the full study cohort, shown as the mean ± standard deviation after subject-level hourly profile aggregation as described in the “Methods” section.

Figure 1 shows 24-h SpO_2_ profiles (mean ± 99.5% confidence interval) stratified by decade of age, BMI group, gender, and location-inferred home altitude. All subject groups exhibited systematic 24-h variation in SpO_2_ with lowest mean values occurring during nocturnal hours (nadir approximately 01:00 local time), and highest mean values occurring during mid-day hours (zenith approximately 11:00). The general cohort (Fig. 2) exhibited a mean diurnal range of approximately 1% saturation. Subject groups having lower mean daytime SpO_2_ tended to yield a larger mean 24-h range of SpO_2_ and disproportionately lower nocturnal SpO_2_, examples of which can be observed for older subject groups (Fig. 1a) and for subjects residing at >1000 m altitude (Fig. 1c).

### Daytime and nocturnal SpO_2_ variation with subject age, BMI, and home altitude

**Fig. 3:**
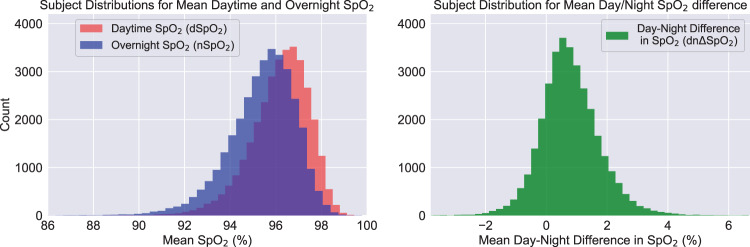
Population distributions of measured mean oxygen saturation values. Separate distributions are shown for daytime mean saturation (dSpO_2_, left panel), nocturnal mean saturation (nSpO_2_, left panel) and mean day-night SpO_2_ difference (dnΔSpO_2_, right panel) for the full study cohort. Positive values for dnΔSpO_2_ correspond to lower measured SpO_2_ at night.

**Fig. 4:**
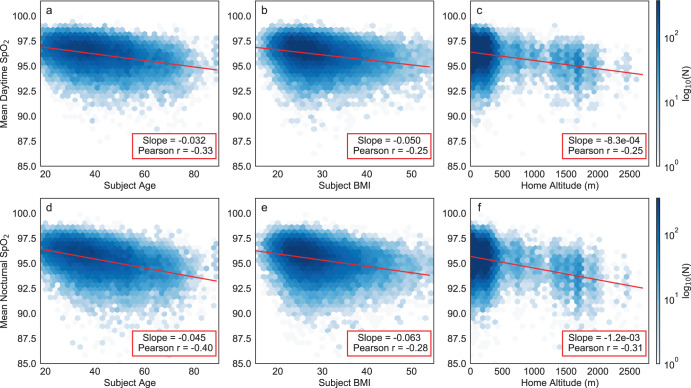
Linear relationships between measured mean oxygen saturation values and each of three independent variables exhibiting the strongest correlation with these metrics. **a**–**c** (top row) correspond to mean daytime SpO_2_. **d**–**f** (bottom row) correspond to mean nocturnal SpO_2_. Independent variables consist of: **a**, **d** age, **b**, **e** body mass index, **c**, **f** home altitude. Each plot presents a 2-dimensional histogram of values from all 33,080 subjects in evenly spaced hexagonal bins, with the color density corresponding to log-scaled bin counts for visual clarity. In each plot, the overlaid red line represents the simple univariate linear regression fit using the independent variable shown on the *x*-axis. The listed slope and Pearson correlation coefficient correspond to the same univariate linear fit.

Histograms of dSpO_2_ and nSpO_2_ are shown in Fig. 3. In the full cohort, mean dSpO_2_ was 96.17 [SD 1.28]%; mean nSpO_2_ was 95.38 [SD 1.47]%; and mean dnΔSpO_2_ was 0.78 [SD 0.98]%. Both dSpO_2_ and nSpO_2_ were significantly correlated with age, BMI, and altitude, and exhibited a monotonic decreasing trend with each of these variables. Figure 4 shows 2D histograms for these metrics overlaid with the corresponding univariate linear regression line, slope and Pearson correlation coefficient. In all cases, both the absolute slope and the correlation coefficients were greater for nSpO_2_ than for dSpO_2_. For daytime SpO_2_, measured slopes with respect to each of these variables were in good quantitative agreement with existing publications^3,8,9^. Table 1 compares the slopes and intercepts for simple univariate regression of daytime SpO_2_ using only age as the independent variable (for subjects with home altitude below the study median of 155m) with the equivalent low-altitude univariate model reported by Crapo et al.^3^.

**Table 1:** Tabulated comparison of reference SaO_2_ linear model coefficients (top row of coefficients; adapted from ref. ^3^) with results obtained from equivalent fits to daytime mean SpO_2_ (bottom row of coefficients) using only low-altitude subjects (estimated home altitude < 155m) from our data set.

Univariate regression results for blood oxygen saturation (low-altitude subjects only)				
Model	*R*^2^	SEE	Constant	Age (years)
Reference univariate SaO_2_ (Crapo et al.^3^)	0.32	0.85	97.66	−0.0296
Daytime SpO_2_ (low-alt. subjects)	0.12	1.14	97.65 (97.57, 97.74)) [*p* < 1.0e−10]	−0.0322 (−0.0341, −0.0303)) [*p* < 1.0e−10]

Values in parentheses indicate 99.5% confidence intervals for fit coefficients. Values in brackets are regression coefficient *p* values, corresponding to two-sided *t* tests under the null hypothesis that the coefficient is equal to zero.

*R*^*2*^ coefficient of determination, *SEE* standard error of the estimate.

Linear regression results for model M_*Ref*_ fit to both daytime and nocturnal SpO_2_ using the full subject cohort are summarized and compared with the reference results reported by Crapo et al. in Table 2. For daytime SpO_2_ the fitted constant term (89.25%, 99.5% CI 88.68–89.83) differs by less than 0.2% saturation compared with the constant term from the reference SaO_2_ model. The value of this constant term is not physiologically interpretable as it corresponds to the predicted oxygen saturation at zero age, weight, and barometric pressure, but instead provides an indication of absolute calibration agreement between the SaO_2_ and SpO_2_ sensors used by the two studies. For daytime SpO_2_ the model coefficients for age, weight, and barometric pressure all have 5–20% smaller absolute magnitude compared with the corresponding reference model coefficients. In contrast, for nocturnal SpO_2_ the fitted model coefficients have 5–20% greater magnitude compared with reference model coefficients. Additionally, on our data set the nocturnal SpO_2_ model fit yields a statistically significant term for sex (0.16% higher nSpO_2_ for males, 99.5% CI 0.11–0.22, *p* = 2.7 × 10^−16^), and both daytime and nocturnal SpO_2_ model fits yield statistically significant coefficients for height, in contrast to the published reference SaO_2_ model which did not report significant fit coefficients for sex or height.

**Table 2:** Tabulated comparison of reference SaO_2_ linear model coefficients (top row of coefficients; adapted from reference^3^) with linear model coefficients obtained from equivalent fits to daytime mean SpO_2_ (middle row of coefficients) and overnight SpO_2_ (bottom row of coefficients) using our data.

Linear regression results for blood oxygen saturation: fit coefficients for reference model (M_*Ref*_)								
Model	*R*^2^	SEE	Constant	Age (years)	Baro. Press. (mm Hg)	Weight (kg)	Sex (*m* = 1, *f* = 0)	Height (cm)
Reference SaO_2_ (Crapo et al.^3^)	0.56	0.85	89.41	−0.0362	0.0128	−0.0159	n.s.	n.s.
Daytime SpO_2_	0.23	1.13	89.25 (88.68, 89.83) [*p* < 1.0e−10]	−0.0309 (−0.0322, −0.0296) [*p* < 1.0e−10]	0.0100 (0.0094, 0.0105) [*p* < 1.0e−10]	−0.0149 (−0.0157, −0.0140) [*p* < 1.0e−10]	−0.0168 (−0.0686, 0.0351) [*p* = 0.36]	0.0123 (0.0098, 0.0149) [*p* < 1.0e−10]
Nocturnal SpO_2_	0.32	1.21	87.12 (86.50, 87.74) [*p* < 1.0e−10]	−0.0431 (−0.0445, −0.0417) [*p* < 1.0e−10]	0.0140 (0.0134, 0.0146) [*p* < 1.0e−10]	−0.0182 (−0.0192, −0.0173) [*p* < 1.0e−10]	0.1629 (0.1071, 0.2187) [*p* < 1.0e−10]	0.0069 (0.0042, 0.0097) [*p* < 1.0e−10]

Values in parentheses indicate 99.5% confidence intervals for fit coefficients. Values in brackets are regression coefficient *p* values, corresponding to two-sided *t* tests under the null hypothesis that the coefficient is equal to zero.

*R*^*2*^ coefficient of determination, *SEE* standard error of the estimate, *n.s.* not significant.

Linear regression results for model M_1_ fit to the full subject cohort for dSpO_2_ and nSpO_2_ are listed in Table 3. For both dSpO_2_ and nSpO_2_, all M_1_ regression coefficients for age, BMI, and home altitude are highly significant. For nocturnal SpO_2_, no coefficients corresponding to categorical variables were identified as significant. However, M_1_ fit using daytime SpO_2_ produced significant coefficient for sex (0.05% higher SpO_2_ for females, 99.5% CI 0.01–0.09, *p* = 4.1 × 10^−4^), Asian race/ethnicity compared with White race/ethnicity (0.10% higher SpO_2_ for White participants, 99.5% CI 0.03–0.17, *p* = 1.2 × 10^−4^), and for Hispanic race/ethnicity compared with White race/ethnicity (0.13% higher SpO_2_ for White participants, 99.5% CI 0.07–0.19, *p* = 4.8 × 10^−10^).

**Table 3:** Linear model M_1_ full-cohort fit results for daytime SpO_2_ (top row of coefficients) and nocturnal SpO_2_ (bottom row of coefficients).

Linear regression results for proposed model (M_1_)											
Model	*R*^2^	SEE	Constant	Age (years)	BMI (kg/m^2^)	Altitude (km)	Sex (*m* = 1, *f* = 0)	Asian race/ethnicity	Black race/ethnicity	Hispanic race/ethnicity	Other race/ethnicity
Full-cohort daytime SpO_2_	0.23	1.12	96.66 (96.62, 96.70) [*p* < 1e−10]	−0.0314 (−0.0327, −0.0300) [*p* < 1e−10]	−0.0457 (−0.0484, −0.0430) [*p* < 1e−10]	−0.8402 (−0.8845, −0.7959) [*p* < 1e−10]	−0.0490 (−0.0880, −0.0101) [*p* = 4.1e−04]	−0.1004 (−0.1735, −0.0273) [*p* = 1.2e−04]	−0.0648 (−0.1446, 0.0150) [*p* = 0.023]	−0.1334 (−0.1936, −0.0733) [*p* = 4.8e−10]	−0.0146 (−0.0986, 0.0694) [*p* = 0.63]
Full-cohort nocturnal SpO_2_	0.32	1.21	95.95 (95.91, 95.99) [*p* < 1e−10]	−0.0429 (−0.0444, −0.0415) [*p* < 1e−10]	−0.0562 (−0.0591, −0.0533) [*p* < 1e−10]	−1.1736 (−1.2214, −1.1257) [*p* < 1e−10]	−0.0038 (−0.0459, 0.0383) [*p* = 0.80]	0.0775 (−0.0015, 0.1564) [*p* = 0.0059]	−0.0068 (−0.0930, 0.0794) [*p* = 0.82]	0.0243 (−0.0406, 0.0893) [*p* = 0.29]	0.0596 (−0.0311, 0.1504) [*p* = 0.065]

Sex variables for the full subject cohort are encoded using a value of 1 for male subjects and 0 for female subjects. Race/ethnicity variables for the full cohort and both sexes encoded using a value of 1 for each listed race/ethnicity group, with White subjects encoded using all zeros. Values listed in parentheses represent 99.5% confidence intervals for the fitted model coefficients. Values in brackets are regression coefficient *p* values, corresponding to two-sided *t* tests under the null hypothesis that the coefficient is equal to zero.

*R*^*2*^ coefficient of determination, *SEE* standard error of the estimate.

### Regression models stratified by sex and race/ethnicity

**Fig. 5:**
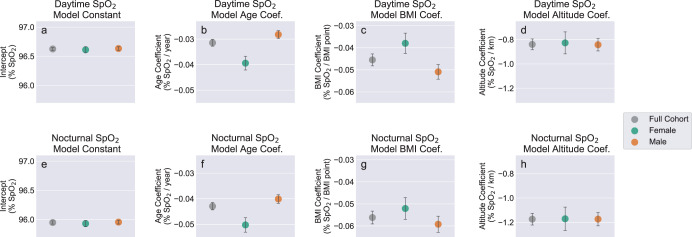
Comparison of fit coefficients for M_1_ models fit to the full cohort and M_1,*sex*_ models fit independently to female and male subjects. **a**–**d** (top row) correspond to models fit using mean daytime SpO_2_. **e**–**h** (bottom row) correspond to models fit using mean nocturnal SpO_2_. Regression coefficients consist of: **a**, **e** constant term, **b**, **f** age, **c**, **g** body mass index, **d**, **h** home altitude. Error bars represent 99.5% confidence intervals for the fitted coefficients. Race/ethnicity variables are omitted for clarity. Plotted coefficients and confidence intervals are identical to the values listed in Supplementary Table 5.

For the purposes of sex-specific stratified analysis, we compared M_1,*sex*_ models fit for male and female subjects separately with M_1_ models fit for full cohort. The fitted model coefficients and confidence intervals are plotted in Fig. 5 to facilitate visual comparison, with the results also tabulated in Supplementary Table 5. For both sexes as well as the full subject cohort, coefficients of determination (*R*^2^) were higher for models fit to nSpO_2_ compared with dSpO_2_. Additionally the fitted model coefficients for age, BMI, and altitude variables all exhibited significantly larger absolute magnitudes for nSpO_2_ compared to dSpO_2_ (implying a greater impact on SpO_2_ from each these variables at night). This phenomenon of greater impact on SpO_2_ from each of these variables overnight is also observable in the grouped 24-h mean profiles shown in Fig. 1a–c, in which the separation between stratified 24-h profiles is consistently larger during nocturnal hours.

Comparing female- and male-specific models shows no meaningful differences for constant terms and altitude coefficients between sexes, in either dSpO_2_ models (Fig. 5a, d) or nSpO_2_ models (Fig. 5e, h). However, the coefficients for age differ significantly between the sex-specific models for both dSpO_2_ (*p* = 1.4 × 10^−24^, Fig. 5b) and nSpO_2_ (*p* = 4.5 × 10^−18^, Fig. 5f), with females producing a larger magnitude for age coefficients (implying greater decline in SpO_2_ with age) for both measurement periods. Additionally, BMI coefficients also differ significantly between sex-specific models for both dSpO_2_ (*p* = 1.1 × 10^−3^, Fig. 5c) and nSpO_2_ (p = 1.9 × 10^−10^, Fig. 5g), with males producing a larger coefficient magnitude (implying greater decline in SpO_2_ with increasing BMI).

**Fig. 6:**
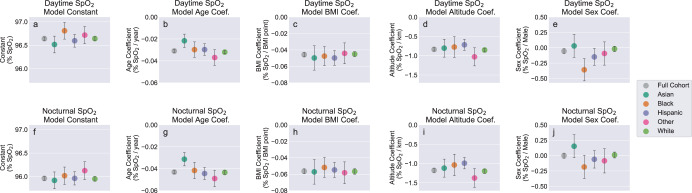
Comparison of model coefficients for for M_1_ models fit to the full cohort, and for M_1,*race*−*ethn*._ models fit independently to each race/ethnicity group. **a**–**e** (top row) correspond to models fit using mean daytime SpO_2_. **f**–**j** (bottom row) correspond to models fit using mean nocturnal SpO_2_. Regression coefficients consist of: **a**, **f** constant term, **b**, **g** age, **c**, **h** body mass index, **d**, **i** home altitude, **e**, **j** assigned sex. Error bars represent 99.5% confidence intervals for the fitted coefficients. Plotted coefficients and confidence intervals are identical to the values listed in Supplementary Table 6.

For additional subgroup analysis we fit model M_1,*race*−*ethn*._ separately for subjects in each of the five race/ethnicity groups reported in the study demographics (Table 4). All race/ethnicity subgroup regression results are plotted in Fig. 6 to facilitate visual comparison, with results tabulated in Supplementary Table 6. Comparing regression coefficients between subgroup models using Welch’s unequal variances *t* test, and employing the Bonferroni-corrected *p* value threshold of 0.0005 to determine statistical significance, identified the following significant pairwise coefficient differences (*p* values for significance tests are shown; individual coefficient values and confidence intervals are listed in Supplementary Table 6):

- Age coefficients (daytime SpO_2_): Significant group differences for Asian participants compared with Other participants (*p* = 4.7 × 10^−6^), and for Asian compared with White participants (*p* = 3.7 × 10^−6^).
- Age coefficients (nocturnal SpO_2_): Significant group differences for Asian compared with Hispanic participants (*p* = 7.6 × 10^−6^), for Asian compared with Other participants (*p* = 2.7 × 10^−7^), and for Asian compared with White participants (*p* = 9.2 × 10^−8^).
- Altitude coefficients (nocturnal SpO_2_): Significant group differences for Hispanic compared with Other participants (*p* = 1.6 × 10^−4^), and for Hispanic compared with White participants (*p* = 2.4 × 10^−4^).
- Sex coefficients (daytime SpO_2_): Significant group differences for Asian compared with Black participants (*p* = 2.8 × 10^−5^), and for Black compared with White participants (*p* = 3.0 × 10^−7^).
- Sex coefficients (nocturnal SpO_2_: Significant group differences for Asian compared with Black participants (*p* = 3.8 × 10^−4^).

All other pairwise group comparisons, including all comparisons for constant terms and BMI coefficients, were not determined to be significant.

**Table 4:** Summary of dataset statistics for the full cohort and demographic groups used for stratified and subgroup analysis.

Subject group	*N* (%)	Age, years (mean ± std. dev.)	BMI, kg/m^2^ (mean ± std. dev.)	Home altitude, m (mean ± std. dev.)	Daytime SpO_2_, % (mean ± std. dev.)	Nocturnal SpO_2_, % (mean ± std. dev.)
Full cohort	33,080 (100.0%)	41.0 ± 13.2	28.9 ± 6.5	260.4 ± 392.9	96.2 ± 1.3	95.4 ± 1.3
Female assigned sex	9169 (27.7%)	40.3 ± 13.1	29.5 ± 7.6	268.6 ± 384.7	96.2 ± 1.4	95.4 ± 1.4
Male assigned sex	23,911 (72.3%)	41.3 ± 13.2	28.7 ± 6.1	257.3 ± 395.9	96.2 ± 1.3	95.4 ± 1.3
Asian race/ethnicity	2063 (6.2%)	37.8 ± 11.8	26.3 ± 4.8	179.2 ± 309.6	96.4 ± 1.2	95.8 ± 1.2
Black race/ethnicity	1687 (5.1%)	39.4 ± 11.8	30.8 ± 7.3	198.3 ± 311.6	96.2 ± 1.3	95.4 ± 1.3
Hispanic race/ethnicity	3162 (9.6%)	36.9 ± 11.1	29.6 ± 6.6	254.9 ± 410.6	96.2 ± 1.3	95.5 ± 1.3
Other race/ethnicity	1501 (4.5%)	39.9 ± 12.0	29.3 ± 6.8	244.1 ± 366.6	96.2 ± 1.4	95.5 ± 1.4
White race/ethnicity	24,667 (74.6%)	42.0 ± 13.5	28.8 ± 6.5	273.2 ± 401.9	96.2 ± 1.3	95.3 ± 1.3

## Discussion

All subject groups in our data set exhibited diurnal variation with similar circadian profiles, consisting of nadir during typical overnight sleep hours and zenith in mid-day (Figs. 1 and 2). Few prior studies have examined systematic 24-h circadian variation in oxygen saturation for healthy adult individuals under naturalistic conditions. Existing studies examining overnight SpO_2_ compared with daytime awake SpO_2_ have typically focused on cohorts presenting with a chronic cardiopulmonary disease such as COPD or sleep apnea. However, findings reported in existing publications regarding circadian and diurnal variation in blood oxygen saturation are in general agreement with both the scale and phase of SpO_2_ variation observed in our data set. A study of 77 healthy pediatric subjects from whom SpO_2_ values were collected at 2-h cadence for 24 h reported systematic sinusoidal variation having an average amplitude of 2% saturation, with lowest values during mid-sleep and highest values in early afternoon hours^21^. Similarly, a study of diurnal variation in arterial oxygen saturation among 22 healthy young adult individuals (mean age 20 years) living at 2600m altitude found that lowest values consistently occurred between hours 01:00 and 03:00^22^. Circadian variation independent of sleep status has also been reported for pulmonary function metrics measured from healthy subjects under controlled conditions, with lowest measured pulmonary function occurring typical sleep hours even while subjects remained awake^23,24^. Combined with prior studies of both healthy individuals and individuals with chronic pulmonary disease which reported no significant differences arterial oxygen pressures for sitting vs. standing and supine positions^25^, this suggests that the lower mean SpO_2_ observed during nocturnal hours is driven primarily by endogenous variation in cardiopulmonary parameters in concert with sleep/wake cycle, rather than by typical recumbent body positions during sleep.

For linear regression models fit to the full subject cohort and for specific subject groups (Table 3 and Supplementary Tables 5 and 6) we have measured consistently stronger effects from age, BMI, and altitude (as well as higher coefficients of determination) for nocturnal SpO_2_ values compared with daytime SpO_2_ values. These phenomena are not specific to the two time windows we have chosen to define daytime and nocturnal measurement periods, but occur consistently for clock hours typically associated with sleep vs. waking and transitional periods (illustrated in Supplementary Fig. 3). The larger effect size at night for these systematic drivers of SpO_2_, combined with the superior model fits for nocturnal SpO_2_, suggests that sleeping conditions provide the best opportunity to resolve meaningful physiological differences as well as avoid potential confounds due to daytime behavior.

Additionally, as can be observed in 24-h mean SpO_2_ profiles for various cohorts (Fig. 1), subgroups with lower daytime SpO_2_ also tend to exhibit a greater decline in SpO_2_ during overnight hours. The three independent variables that most strongly influence daytime and nocturnal SpO_2_ (age, BMI, and altitude) are also significant predictors of the change in SpO_2_ from day to night (dnΔSpO_2_). The correlation between dnΔSpO_2_ and these three independent variables is illustrated in Supplementary Fig. 6. Identifying and quantifying additional unexplained factors driving systematic nocturnal changes in SpO_2_ (specifically instances with overnight decline) merits further investigation.

The age-dependent average decline in oxygen saturation measured for the full subject cohort (−0.031%/year for dSpO_2_) is in close quantitative agreement with trends published previously by other researchers (−0.036%/year reported by Crapo et al.^3^, −0.027%/year reported by Perez-Padilla et al.^20^, −0.020%/year reported by Klæstrup, et al.^19^). Progressive decline in pulmonary function with age has been described extensively in research literature, with quantitative trends reported for spirometry metrics, respiratory muscle function, gas exchange metrics, and physical lung tissue properties such as elastic recoil and alveolar size^26^. Age-related lung tissue changes include progressive remodeling of the collagen fibers that surround and support the alveoli, contributing to increased average alveolar size and loss of elastic recoil. Combined, this results in a tendency for smaller airways of older lungs to close during breathing even under resting conditions^27^. The closure of these airways translates into mismatch between alveolar ventilation and pulmonary capillary perfusion (V/Q mismatch) which hampers the diffusion of inhaled oxygen into the arterial blood stream^28^. Additionally, alveolar enlargement reduces total alveolar surface area, which further impairs gas exchange and contributes to increasing alveolar-arterial O_2_ gradient^26^. Collectively these age-related changes cause a progressive decline in arterial oxygen saturation that is approximately linear with age, even in the absence of overt lung disease^3,19,29^.

Our findings regarding the continuous linear relationship between increasing body weight and decreasing arterial oxygen saturation (measurable even between non-obese BMI categories) is in close quantitative agreement with prior published work. The linear regression model for daytime SpO_2_ fit to the full subject cohort in our data set (Table 3) yields a slope of −0.046%/BMI-point for dSpO_2_, compared with −0.036%/BMI-point reported by Perez-Padilla et al.^20^.

Body weight-associated changes in pulmonary function and arterial oxygen saturation have been studied most commonly in the context of severe obesity (BMI > 40)^30–33^, although some published research has reported significant trends in spirometry metrics as a function of BMI even for normal and overweight (non-obese) categories^34–37^. Researchers have consistently reported a negative correlation between arterial oxygen saturation and BMI or weight, even in the absence of obstruction or pulmonary co-morbidities. The hypothesized mechanisms of interaction between body composition and pulmonary function include both direct mechanical effects such as lung unit closure and atelactasis (reducing functional lung capacity, and increasing V/Q mismatch), as well as adiposity-mediated pulmonary tissue inflammation^38,39^. Further, these obesity-related effects on pulmonary function and oxygen saturation are expected to have a greater impact during nocturnal sleep hours compared with awake daytime hours^40^, which may explain the slightly larger effect size for BMI we have measured for nSpO_2_ vs. dSpO_2_ in the full cohort and all subject groups (−0.046%/BMI-point for dSpO_2_ vs. −0.056%/BMI-point for nSpO_2_ fit using the full subject cohort).

The sex-specific regression models (summarized in Fig. 5 and Supplementary Table 5) support two conclusions regarding systematic differences in SpO_2_ trends between sexes. First, SpO_2_ tends to decline more rapidly with increasing BMI for males than females. Additionally, SpO_2_ tends to decline more rapidly with increasing age for females than males. Although some existing blood oxygen saturation studies have reported small but significant relationships between measured SpO_2_ and female sex (exclusively as additive sex-specific offsets^5,10,11^), to our knowledge no prior published work has quantified differing sex-dependent trends for age and BMI.

The sex-specific difference in SpO_2_ trend vs. BMI (Fig. 5c) may be attributable to systematic variation in body fat distribution between males and females. Males tend to have disproportionately higher abdominal and visceral adipose tissue than females, even accounting for BMI and total body fat percentage^41^. In light of the reported inverse relationships between abdominal body fat and pulmonary function^30,32^, the disproportionate accumulation of abdominal and visceral body fat among males may explain the greater decrease in SpO_2_ with each incremental increase in BMI.

The significant sex-dependent trends for SpO_2_ vs. age observed in our data (Fig. 5b, f) have not been reported previously among healthy cohorts. However, some pathological lung conditions such as asthma, COPD and pulmonary hypertension display prevalence trends that vary with sex, potentially mediated through the influence of sex hormones on lung function^42^. The transition from regular menstrual status to post-menopause is associated with acceleration of age-related decline in lung function, as quantified by lung capacity metrics such as forced vital capacity^43^. Additionally, sex-dependent differences in age-related trends have been reported for some cardiovascular metrics including blood pressure^44,45^.

In conjunction with the significant difference in age-related SpO_2_ trends between males and females, it is also important to note that the constant terms do not differ statistically between the sexes. Controlling for altitude and BMI, sex-specific differences are small or negligible for young individuals, although with advancing age mean SpO_2_ declines faster for females than males. This effect can be observed visually in Fig. 1d, which overlays male and female mean 24-h SpO_2_ profiles for moderate-BMI, low-altitude subject groups of two different age strata (20−30 years and 60–70 years). In the younger age group males and females present nearly equal mean SpO_2_ across all hours of the day, however for older ages the SpO_2_ profiles diverge and females exhibit lower SpO_2_ across all hours.

The decline in SpO_2_ with increasing altitude is well-established, and occurs as a direct result of the reduced oxygen partial pressure in the ambient environment. Because of the nonlinear (though monotonic) relationship between altitude and mean atmospheric pressure^46^, and the sigmoid shape of the oxygen-hemoglobin dissociation curve^47^, the theoretical trend for arterial oxygen saturation with altitude is not expected to be perfectly linear. However, significant deviation from a consistent linear trend only occurs at high altitudes (>2500 m)^48^, and therefore for altitude range evaluated in our data set a linear approximation is adequate.

Given the optical basis for the function of pulse oximeter devices, which employ both infrared and visible wavelengths of light, many researchers and clinicians have raised valid concerns regarding the accuracy of pulse oximetry measurements across the full spectrum of human skin tone. Three recent studies utilizing large hospital-gathered data sets consisting of opportunistic paired SpO_2_ and arterial blood gas measurements have reported significant differences in SpO_2_ measurement accuracy depending on patient race and ethnicity at low oxygen saturation values^49–52^. These inaccuracies among in-hospital SpO_2_ measurements disproportionately impact patients of non-White race/ethnicity^50^, particularly Black individuals^49,51,52^.

**Fig. 7:**
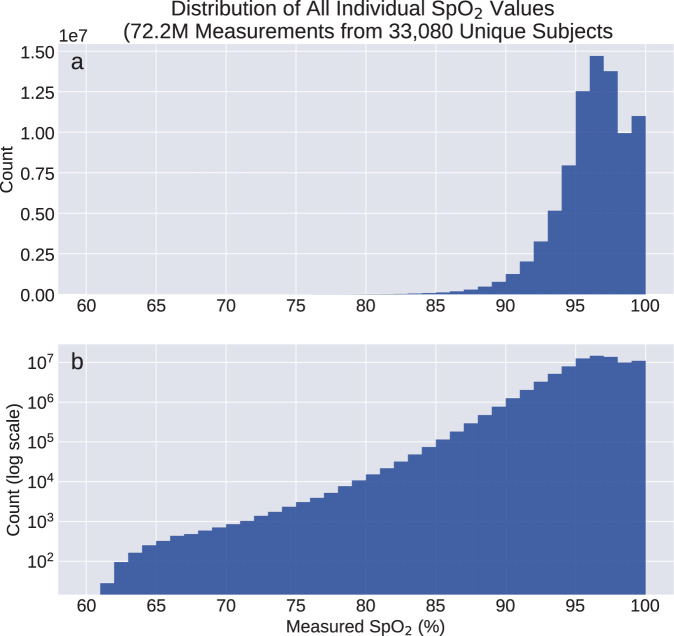
Histograms of all individual SpO_2_ values collected from the full subject cohort. **a** Distribution counts shown with linear y-scale. **b** Distribution counts shown with logarithmic *y*-scale. The two histograms represent identical data, but with differing *y*-scales to enable useful visualization of the value distribution for SpO_2_ < 85%.

On the data set reported here, stratified analysis according to self-reported race/ethnicity (Fig. 6 and Supplementary Table 6) does not indicate the presence of any significant or meaningful systematic bias in SpO_2_ measurements between race/ethnicity groups. For both dSpO_2_ and nSpO_2_, regression models fit to subjects of each race/ethnicity group yield constant terms with no significant differences between groups (Fig. 6a, e). Additionally, for regression models incorporating categorical variables encoding each race/ethnicity group fit to female subjects, male subjects, and the full subject cohort (rightmost four columns of Table 3 and Supplementary Table 5, race/ethnicity coefficients correspond to differences smaller than ±0.15% saturation between White and non-White subject groups in our dataset. Combined, this suggests the absence of a clinically meaningful SpO_2_ measurement bias with skin tone over the range of saturation values collected in this study. However, because this data set consists of nominally healthy individuals outside of clinical settings, the range of measured SpO_2_ values is heavily weighted toward non-hypoxic conditions. Just 2.5% of all collected SpO_2_ values fall below 90% saturation, and 0.29% fall below 85% saturation (Fig. 7). Therefore using this data set we are not able to confirm or refute the systematic race/ethnicity differences reported from clinical SpO_2_ data sets that include hypoxic values^49–51^.

For further inspection of differences in mean SpO_2_ according to race/ethnicity, we also compared dSpO_2_ and nSpO_2_ distributions by race/ethnicity group after linear adjustment all individual data points (using sex-specific regression model fits) to correspond to subject age of 40 years, BMI of 25.0, and sea level home altitude. The resulting distributions show no statistically significant differences between race/ethnicity groups based on two-sample Kolmogorov–Smirnov tests, either over the full SpO_2_ range or if the distributions are clipped at 94% saturation to emphasize the hypoxic SpO_2_ range. An example comparison of adjusted nSpO_2_ distributions for Black and White subjects is shown in Supplementary Fig. 7.

**Fig. 8:**
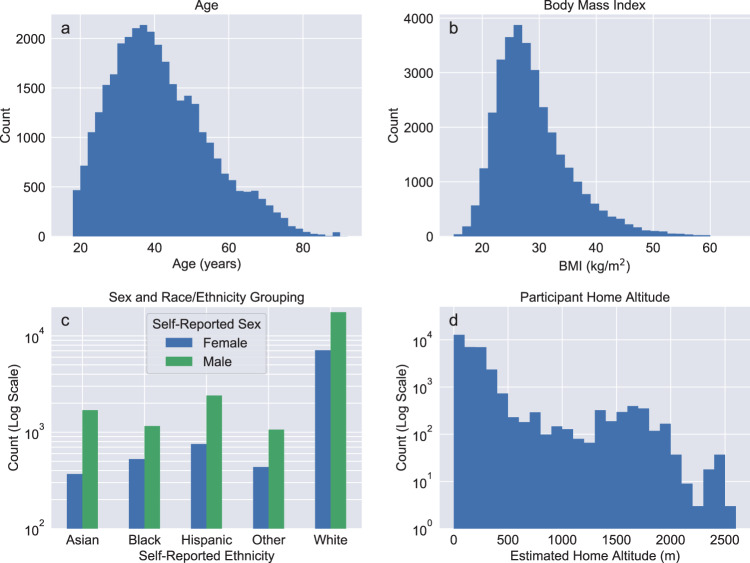
Demographic variable distributions for all subjects used in the analysis. **a** Age, **b** BMI, **c** sex and race/ethnicity, **d** home altitude. Note that for age and BMI the *y*-axis representing subject counts uses a linear scale, while for sex and race/ethnicity groups and estimated home altitude the *y*-axis uses logarithmic scale for clarity.

This study has several important limitations. Although the Apple Heart & Movement Study represents a large total subject pool, it contains significant demographic imbalances as illustrated in Fig. 8d and Table 4. For example, 53% of the cohort used in the analysis reported here is White and male. Additionally all subject metadata including age, body measurements, geographic location (from which elevation and barometric pressure are inferred), sex, and race/ethnicity have been provided by subject self-report without independent verification.

This study did not exclude any subjects based upon cardiovascular or pulmonary disease risk factors, behavior (including alcohol and smoking habits), or self-reported chronic health conditions that may significantly impact blood oxygen saturation (such as COPD, emphysema, sleep apnea, and heart failure). However, regression modeling on subjects stratified according to self-reported health conditions and smoking habits indicates that systematic decline in SpO_2_ with Age and BMI occurs at rates that are similar for healthy lifetime nonsmokers as well as individuals who smoke or have chronic cardiopulmonary conditions (results summarized in Supplementary Note 5, Supplementary Fig. 5, and Supplementary Table 11).

The study period also occurred in the midst of the COVID-19 pandemic (spanning the timeframe when vaccination became widely available in the US), during which an unknown fraction of the study population may have experienced acute respiratory infection. As such, the aggregated data inevitably includes some measurements collected under pathological conditions and this may influence the resulting population-scale observations and statistical models. Additionally, reducing the data collection window to a maximum of 30 consecutive calendar days per subject did not meaningfully impact the downstream regression modeling results (results summarized in Supplementary Note 4, Supplementary Fig. 4, and Supplementary Table 10).

All data in the study was collected in uncontrolled naturalistic conditions, and therefore contains a large variety of unknown measurement contexts and use conditions which may influence the measured SpO_2_ values. Additionally, the grouping of measurements into nocturnal vs. daytime average values is determined by referencing against local clock time, as opposed to grouping according to subject-specific physiological measures such as sleep or activity state. This grouping likely introduces a mix of both awake and asleep measurements into each subject’s dSpO_2_ and nSpO_2_ values. However, fitting and comparing linear model coefficients for individual clock hours does not reveal significant variability between adjacent hours, but rather a smooth circadian variation for each coefficient value (results shown in Supplementary Fig. 3). Additional analysis utilizing sleep tracking data from a subset of subjects to align SpO_2_ measurements with circadian sleep/wake schedule did not meaningfully impact the downstream regression modeling results (results summarized in Supplementary Note 3, Supplementary Figs. 1 and 2, and Supplementary Table 9).

Lastly, because the AppleWatch Series 6 sensor is not a CO-oximeter it is unable to measure or account for the presence of non-oxygen-carrying dyshemoglobin compounds such as carboxyhemoglobin (which may be increased due to use of cigarettes and exposure to other smoke sources), sulfhemoglobin, and methemoglobin.

## Methods

### Data collection

This study examined data from the Apple Heart and Movement Study, an ongoing research study beginning November 14, 2019 conducted in partnership with American Heart Association and Brigham and Women’s Hospital that was designed to explore the links between physical activity and cardiovascular health. Study participants were all Apple Watch users at least 18 years old residing in the United States, and provided informed consent electronically in the Apple Research app. The study was approved by the Advarra Central Institutional Review Board, and registered to ClinicalTrials.gov (ClinicalTrials.gov Identifier: NCT04198194)^53^. All data collection, both raw measurements and metadata, was accomplished using the using the Apple Research app.

**Fig. 9:**
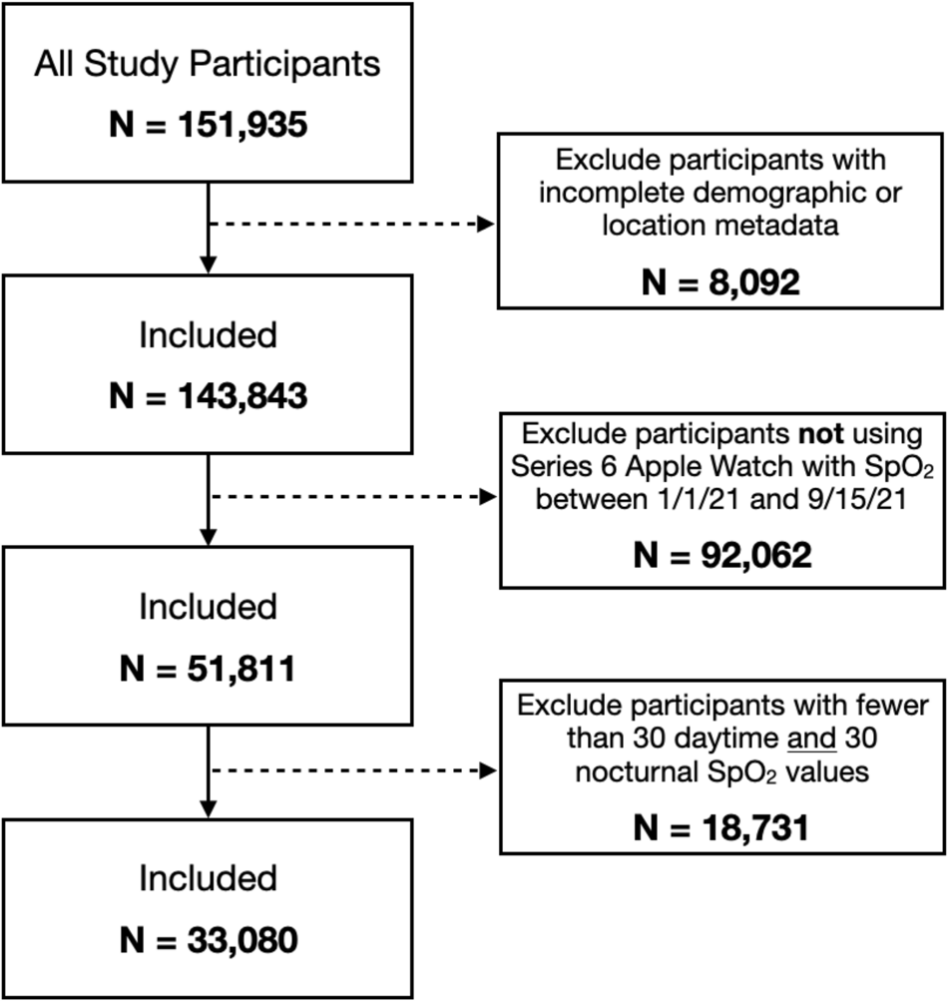
Subject inclusion/exclusion criteria flowchart.

Subjects were selected for inclusion in downstream analysis based on use of a Series 6 Apple Watch and contribution of sufficient SpO_2_ measurements during the study period as described in the flowchart in Fig. 9. Subject demographic distributions including age, body mass index, estimated home altitude, and self-reported race and ethnicity are summarized in Fig. 8. Geographic information was based on zip (postal) code, with 3-5 zip code digits available depending on total participant count in that location (for privacy purposes, zip codes containing few subjects were reported with the trailing two digits redacted). Approximate home altitude was determined by associating the zip code information with USGS mean surface elevation in the corresponding geographic area. Due to comparatively small numbers of individuals self-reporting ethnicity of “American Indian or Alaskan Native,” “Middle Eastern or North African,” “Native Hawaiian or other Pacific Islander,” and “None of these fully describes me,” these subjects were combined into a single race/ethnicity group (Other) when used for downstream subgroup and stratified analysis. Body mass index was determined from height and weight, and mean barometric pressure was calculated from home altitude using the reference NOAA Pressure Altitude equation^46^. Tabulated summary statistics and statistical comparisons are shown in Table 4.

All individual SpO_2_ measurements from Series 6 Watches collected between January 1, 2021 and September 15, 2021 were aggregated from active study participants, along with self-reported demographic information. Blood oxygen saturation values were measured using the Apple-developed SpO_2_ sensor available on some Apple Watch models (only data from Apple Watch Series 6 devices is utilized in the present study). SpO_2_ values were acquired both on-demand (initiated by the watch wearer) as well as passively via background measurements attempted automatically under low-motion conditions at roughly 30-min cadence. Histograms of all individual SpO_2_ values (ranging from 60 to 100% saturation with integer values) collected from the full study cohort are shown in Fig. 7. The Apple Watch SpO_2_ measurement accuracy compared against reference clinical fingertip pulse oximeters has been reported elsewhere for healthy subject cohorts (*N* = 265 subjects, mean bias −0.23%, 95% limits of agreement −3.49% to 3.04% compared with Nellcor PM10N Oximeter reference^54^) and cohorts enriched with cardiopulmonary disease (*N* = 100 subjects, mean bias 0.8%, 95% limits of agreement −2.7% to 4.1% compared with Mobil POD-2 Finger Oximeter and Multilaser OX-06 Oximeter references^55^).

Individual SpO_2_ values were labeled with timestamps corresponding to wall clock time in the subject’s local time zone. All downstream analysis utilized data from subjects contributing at least 30 individual SpO_2_ measurements during typical mid-sleep hours (local wall clock time 01:00–04:59) as well as at least 30 individual SpO_2_ measurements during typical awake daytime hours (local wall clock time 11:00–18:59). For subjects satisfying these selection criteria (Fig. 9) all SpO_2_ measurements collected during the study period were retained, with no outlier rejection, thresholding, filtering, or other removal of individual SpO_2_ values. This data aggregation yielded 33,080 unique subjects contributing over 72.2 million individual SpO_2_ values (median 1772 values/subject) spanning all hours of the day. A complete dataset for each subject consisted of mean daytime and nocturnal SpO_2_, approximate home altitude inferred from zip code information, and self-reported age, assigned sex, height, weight, and race/ethnicity.

### Data processing

Individual SpO_2_ values from each subject were grouped and averaged by hour of the day, yielding a single 24-h mean SpO_2_ profile per subject, irrespective of the subject’s total number of collected SpO_2_ measurements or their hourly distribution throughout the day. Subject 24-h profiles were then averaged over either the full cohort or various subject groups (for example subjects stratified by decade of age or BMI category). The 24-h SpO_2_ profile mean and standard deviation for the general cohort is shown in Fig. 2, and 24-h SpO_2_ profile means and 99.5% confidence interval profiles for subject groups stratified by age, BMI, home altitude, and sex are shown in Fig. 1. This method for aggregating hourly SpO_2_ values for the full cohort and stratified groups minimizes bias due to the number of individual measurements per subject, and has been reported in prior literature for circadian analysis of blood pressure profiles stratified by various demographic variables^45^.

Per-subject mean daytime oxygen saturation (dSpO_2_) and mean nocturnal oxygen saturation (nSpO_2_) were calculated for each individual by averaging all SpO_2_ values occurring between 11:00 and 18:59 local clock time, and 01:00–04:59 local clock time, respectively. The hourly time ranges used to define dSpO_2_ and nSpO_2_ were chosen prior to performing downstream statistical analysis on these metrics. Mean day–night oxygen saturation difference (dnΔSpO_2_) for each individual was determined from the difference between these two metrics (dSpO_2_ − nSpO_2_), with positive values of dnΔSpO_2_ corresponding to lower average blood oxygen saturation overnight than during the daytime. Full-cohort distributions of dSpO_2_, nSpO_2_, and dnΔSpO_2_ are shown in Fig. 3.

### Statistical analysis

Plotting and data visualization were performed using the Python packages Seaborn^56^ (version 0.11.0) and Matplotlib^57^ (version 3.2.2). Ordinary least squares linear regression modeling (OLS) was performed using the Python statsmodels module^58^ (version 0.11.1) to quantify systematic factors impacting measured blood oxygen saturation at the population level. Dependent variables consisted of dSpO_2_ and nSpO_2_ separately. Various sets of independent variables were used for fitting linear regression models, with all reported models summarized in Table 5.

**Table 5:** Summary of models employed in linear regression analysis.

Model name	Covariates	Model usage
M_*Age*_	Age (years)	Comparison with low-altitude univariate SaO_2_ model (Crapo, et al.^3^)
M_*Ref*_	Age (years)	Comparison with reference SaO_2_ model (Crapo, et al.^3^)
	Height (cm)	
	Weight (kg)	
	Barometric pressure (mmHg)	
	Sex (categorical)	
M_1_	Age–40 (years)	Proposed full-cohort SpO_2_ linear model
	BMI–25 (kg/m^2^)	
	Home altitude (km)	
	Sex (categorical)	
	Race/ethnicity (categorical)	
M_1,*sex*_	Age–40 (years)	Sex-stratified analysis
	BMI–25 (kg/m^2^)	
	Home altitude (km)	
	Race/ethnicity (categorical)	
M_1,*race*−*ethn*._	Age–40 (years)	Race/ethnicity-stratified analysis
	BMI–25 (kg/m^2^)	
	Home altitude (km)	
	Sex (categorical)	

M_1*,sex*_ differs from M_1_ only by the omission of the categorical variable encoding sex. M_1,*race−ethn*._ differs from M_1_ only by the omission of the categorical variables encoding race/ethnicity.

Analysis of sources of variation in daytime and nocturnal SpO_2_ was accomplished by first calculating dSpO_2_ and nSpO_2_ for each subject on a date-by-date basis, then performing nested one-way ANOVA and variance components analysis (VCA) utilizing mixed-effects modeling as reported in the Supplementary Materials (Supplementary Note 1 and Supplementary Tables 3 and 4). For both daytime and nocturnal SpO_2_, nested ANOVA and VCA both support the conclusion that the predominant contributor to daily measurement variance is subject-to-subject differences.

For direct comparison with the arterial oxygen saturation reference equation reported by Crapo et al.^3^, dSpO_2_ and nSpO_2_ were modeled using a combination of age, height, weight, assigned sex and inferred barometric pressure (estimated from home altitude). This reference model employing height and weight separately in place of BMI, and barometric pressure in place of home altitude, is referred to as M_*Ref*_ in subsequent discussion. For subjects residing at low altitude (below the dataset median of 155m) we fit a simple univariate model (M_*Age*_) for dSpO_2_ using only age as the independent variable, for comparison against the univariate regression model reported by Crapo et al. for low-altitude measurements^3^.

For additional full-cohort analysis we fit dSpO_2_ and nSpO_2_ using model M_1_, which employed linear terms for the following covariates: age, BMI, estimated home altitude, assigned sex (categorically encoded using 1 corresponding to male sex and 0 corresponding to female sex), and self-reported race/ethnicity group (categorically encoded using dummy variables, with ‘White’ race/ethnicity used as the reference category based on greatest subject count in this subject group). Quadratic terms for age, BMI and altitude were evaluated but did not produce models with meaningfully different goodness of fit metrics compared to models using only linear terms, and so were not utilized for further analysis. For all fits using model M_1_, M_1,*sex*_ and M_1,*race*−*ethn*._, the age and BMI covariates were centered at 40 years and 25.0 BMI points, respectively. Estimated home altitude values were used uncentered. The fitted constant terms therefore represent the predicted mean SpO_2_ for an individual residing at sea level with age 40 and BMI of 25.0 points.

In order to evaluate the presence of systematic factors impacting measured SpO_2_ as a function of subject sex, race or ethnicity, we performed the following analysis across subject groups using dSpO_2_ and nSpO_2_ as dependent variables:

- Model coefficients and confidence intervals corresponding to sex and race/ethnicity variables were examined, for the M_1_ model fit to the full subject cohort.
- Stratified regression models were fit separately for male and female participants using model M_1,*sex*_ and the resulting coefficients and confidence intervals were compared between these models.
- Stratified regression models were fit separately for participants in each race/ethnicity group using model M_1,*race*−*ethn*._ and the resulting coefficients and confidence intervals compared between models.

Additional regression models incorporating linear interaction terms for sex and race/ethnicity were investigated, but yielded either inferior goodness of fit metrics (compared with model M_1_) or produced results numerically equivalent to the stratified regression models M_1,*sex*_ and M_1,*race*−*ethn*._. Details of these alternative investigated models are provided in Supplementary Note 2 and Supplementary Tables 7 and 8.

In accordance with recent recommendations regarding use of *p* values in statistical analysis^59^, we have used a threshold of *p* < 0.005 (rather than *p* < 0.05) to determine statistical significance. Correspondingly, we report uncertainty for fitted model coefficients using 99.5% confidence intervals, and all plotted error bars correspond to 99.5% confidence interval. For the grouped circadian SpO2 profiles shown in Fig. 1), error whiskers represent 2.81 times the standard error of the mean (SEM) to reflect 99.5% confidence interval. All *p* values were calculated using the SciPy stats package^60^ (version 1.5.0). *p* Values reported for linear regression coefficients correspond to two-sided *t* tests under the null hypothesis that the coefficient is equal to zero. In stratified analysis, *p* values reported for comparing coefficients between separate linear regression models fit to independent data subsets (for example data from females vs. males) were determined using Welch’s unequal variances *t* test, under the null hypothesis that the two coefficients are equal. For race/ethnicity stratified analysis, when comparing coefficients between models we utilized a Bonferroni-corrected *p* value threshold of <0.0005 to account for multiple pairwise comparisons.

### Reporting summary

Further information on research design is available in the Nature Research Reporting Summary linked to this article.

## Supplementary information

Supplemental Material

Reporting Summary

## Data Availability

The aggregated data that support the findings of this study can be made available on request from the corresponding author (M.O.). Request for data will be evaluated and responded to in a manner consistent with the specific language in the study protocol and informed consent form.

## Code availability

Code for all data analyses and statistical modeling was written in Python 3.6. This code may be available upon request from the corresponding author (M.O.). Requests for code will be evaluated and responded to in a manner consistent with policies intended to protect participant confidentiality and language in the study protocol and informed consent form.
