# Supplementary material for: Pulse oximetry values from 33,080 participants in the Apple Heart & Movement Study
Source: NPJ Digit Med. 2023 Jul 27;6:134. doi: 10.1038/s41746-023-00851-6 (PMC10374661; doi:10.1038/s41746-023-00851-6)
Supplement: Supplementary file 1 — Supplemental Material [file 41746_2023_851_MOESM1_ESM.pdf]

Supplementary Materials: Pulse Oximetry Values from 33,080 Participants in the Apple Heart & Movement Study

Ian Shapiro[1], Jeff Stein[1], Calum MacRae[2,3], and Michael O'Reilly[1,*]

[1]Apple Inc., Cupertino, CA
[2]Cardiovascular Medicine Division, Brigham and Women's Hospital, Boston, MA
[3]Harvard Medical School, Boston, MA
[*]Corresponding author: mor@apple.com

Figure Legends

Supplementary Figure 1: Distribution of all nightly sleep start times relative to midnight (a). Comparison of mean SpO_2 24-hour profile based on alignment by clock time with mean SpO_2 24-hour profile based on alignment by sleep start time for all subjects in the Sleep Cohort (b). Scatterplots of original clock-aligned and sleep-aligned $dSpO_2$ (c) and $nSpO_2$ (d) for all subjects in theSleep Cohort.

Supplementary Figure 2: Comparison of regression coefficients for M_1 models fit using the full cohort data aligned by clock time ('Original Cohort, Aligned by Clock Time'), using the Sleep Cohort aligned by clock time ('Sleep Cohort, Aligned by Clock Time'), and using the Sleep Cohort aligned by sleep tracking measurements ('Sleep Cohort, Aligned by Sleep Time'), for daytime SpO_2 (a-d, top row) and nocturnal SpO_2 (e-h, bottom row). Error bars represent 99.5% confidence intervals for the fitted coefficients. Race/Ethnicity variables are omitted for clarity. Plotted coefficients and confidence intervals are identical to the values listed in Supplementary Table 9.

Supplementary Figure 3: Linear regression R^2 and model coefficients produced by fitting M_1 using subject-mean SpO_2 aggregated for each individual hour of the day: (a) Fitted R^2 is highest during typical sleep hours (approx 22:00–06:00) compared with daytime hours. Age (c), BMI (d) and altitude (f) coefficients exhibit clear circadian variation and have greatest absolute magnitude during typical sleep hours. (e) Biological sex and race/ethnicity group (g-j) also exhibit a small degree of diurnal variation. In all plots, error whiskers correspond to 99.5% confidence intervals.

Supplementary Figure 4: Comparison of regression coefficients for M_1 models fit to the full cohort using SpO_2 measurements from all available dates for each subject ('Original Cohort, Full Study Window'), for a subset of subjects with SpO_2 measurements limited to a maximum timespan of 30 calendar days ('Rolling 30d Cohort, 30d Data Window'), and for the same subset of subjects using SpO_2 measurements from all available dates ('Rolling 30d Cohort, Full Study Window'), for daytime SpO_2 (a-d, top row) and nocturnal SpO_2 (e-h, bottom row). Error bars represent 99.5% confidence intervals for the fitted coefficients. Race/Ethnicity variables are omitted for clarity. Plotted coefficients and confidence intervals are identical to the values listed in Supplementary Table 10.

Supplementary Figure 5: Comparison of regression coefficients for M_1 models fit for subjects stratified by self-reported health conditions and smoking habits, for daytime SpO_2 (a-d, top row) and nocturnal SpO_2 (e-h, bottom row). Error bars represent 99.5% confidence intervals for the fitted coefficients. Race/Ethnicity variables are omitted for clarity. Plotted coefficients and confidence intervals are identical to the values listed in Supplementary Table 11.

Supplementary Figure 6: Linear relationships between mean day-night SpO_2 difference (dn$\Delta$$SpO_2$) and the three independent variables exhibiting the strongest correlation with these metrics: age (a), BMI (b) and estimated home altitude (c). Each plot presents a 2-dimensional histogram of values from all 33,080 subjects in evenly-spaced hexagonal bins, with the color density corresponding to log-scaled bin counts for clarity. Positive values for dn$\Delta$$SpO_2$ correspond to an overnight drop in measured SpO_2. In each plot, the overlaid red line represents the simple univariate linear regression fit using the independent variable shown on the x-axis. The listed slope and Pearson correlation coefficient correspond to the same univariate linear fit.

Supplementary Figure 7: Histograms of nocturnal SpO_2 for Black and White subjects, after linear adjustment of age to a target of 40 years, BMI to a target of 25.0 kg/m^2, and home altitude to zero elevation. Distributions are shown for the full range of nocturnal SpO_2 (a) and for the low-saturation range (b). Data for both plots is identical, with the only the axes limits differing. The distributions do not differ with statistical significance ($p > .05$) based on two-sample Kolmogorov-Smirnov test using two-sided alternative hypothesis, either over the full range of SpO_2 or if the distributions are clipped at 94% saturation to emphasize the hypoxic tail.

Supplementary Note 1 (Variance Analysis)

To investigate the magnitude of subject-to-subject variability in $dSpO_2$ and $nSpO_2$ compared to day-to-day variability within subjects, we prepared a dataset consisting of per-date $dSpO_2$ and $nSpO_2$ by averaging SpO_2 values in 24-hour time windows for each subject, using the same clock hours as described in the Methods to delineate daytime and nocturnal measurements. We excluded individual subject-dates containing only a single daytime or nocturnal SpO_2 measurement, representing 1.3% of total subject-dates. This yielded an array of 3.41M per-date $dSpO_2$ values and 3.71M per-date $nSpO_2$ values for downstream variance analysis via nested one-way ANOVA and variance components analysis (VCA).

Nested one-way ANOVA results for $dSpO_2$ and $nSpO_2$ are summarized in Supplementary Tables 1 and 2. For both daytime and nocturnal measurements, the between-subjects variance is highly significant (F-test $p < 1.0e\text{-}10$), with η^2 values of 0.365 and 0.539 for $dSpO_2$ and $nSpO_2$, respectively.

Nested ANOVA: Daytime SpO_2						
Source	SS	df	MS	F-statistic	p-value	η^2
Subject	5.392e+06	33079	163.0	58.61	$p < 1.0e\text{-}10$	0.365
Date(Subject)	9.399e+06	3367943	2.781	-	-	-

Supplementary Table 1: Summary of nested ANOVA results for date-by-date $dSpO_2$. The reported P-value corresponds to F-test applied to the F-statistic. SS = sum of squares. df = degrees of freedom. MS = mean square.

Nested ANOVA: Nocturnal SpO_2						
Source	SS	df	MS	F-statistic	p-value	η^2
Subject	7.981e+06	33079	241.3	129.9	$p < 1.0e\text{-}10$	0.539
Date(Subject)	6.830e+06	3678335	1.857	-	-	-

Supplementary Table 2: Summary of nested ANOVA results for date-by-date $nSpO_2$. The reported P-value corresponds to F-test applied to the F-statistic. SS = sum of squares. df = degrees of freedom. MS = mean square.

Using the same set of per-date $dSpO_2$ and $nSpO_2$ values for each subject, we also performed nested VCA using a mixed-effects linear model with random subject intercepts, fitted via random effects maximum likelihood (REML). Models were fit separately for $dSpO_2$ and $nSpO_2$ to yield estimates for variance between subjects, date-by-date variance within subjects, and residual variance. VCA results are summarized in Supplementary Tables 3 and 4.

For both daytime and nocturnal SpO_2, VCA results support the conclusion that the predominant contributor to daily measurement variance is subject-to-subject differences. Results of VCA are in close quantitative agreement with nested ANOVA results regarding the fraction of variance attributed to subject effects. Additionally, the relative variance contribution from subject variance components is greater for nocturnal SpO_2 compared to daytime SpO_2, a finding that is in agreement with our observation of consistently higher linear model fit R^2 values (Tables **??** and **??**, Supplementary Tables 5, 6, and 8) for $nSpO_2$ than for $dSpO_2$.

Variance Components Analysis: Daytime SpO_2		
Component	Variance Estimate	Pct. Total Variance
Subject	1.61	36.6%
Date(Subject)	1.35	30.7%
Residual	1.43	32.6%

Supplementary Table 3: Nested variance components analysis for date-by-date daytime SpO_2.

Variance Components Analysis: Nocturnal SpO_2		
Component	Variance Estimate	Pct. Total Variance
Subject	2.14	53.6%
Date(Subject)	0.73	18.3%
Residual	1.12	28.1%

Supplementary Table 4: Nested variance components analysis for date-by-date nocturnal SpO_2.

Tabulated Regression Results for Models Stratified by Self-Reported Sex and Race/Ethnicity

Linear Regression Results for Proposed Model (M_1) and Sex-Stratified Model ($M_{1,sex}$)

Model	R^2	SEE	Constant	Age (yr)	BMI (kg/m^2)	Altitude (km)	Sex (m=1, f=0)	Asian Race/Ethnicity	Black Race/Ethnicity	Hispanic Race/Ethnicity	Other Race/Ethnicity
Full Cohort Daytime SpO_2	0.23	1.12	96.66<br>(96.62,96.70)<br>[p <1e-10]	-0.0314<br>(-0.0327,-0.0300)<br>[p <1e-10]	-0.0457<br>(-0.0484,-0.0430)<br>[p <1e-10]	-0.8402<br>(-0.8845,-0.7959)<br>[p <1e-10]	-0.0490<br>(-0.0880,-0.0101)<br>[p=4.1e-04]	-0.1004<br>(-0.1735,-0.0273)<br>[p=1.2e-04]	-0.0648<br>(-0.1446,0.0150)<br>[p=0.023]	-0.1334<br>(-0.1936,-0.0733)<br>[p=4.8e-10]	-0.0146<br>(-0.0986,0.0694)<br>[p=0.63]
Female Daytime SpO_2	0.24	1.18	96.61<br>(96.56,96.66)<br>[p <1e-10]	-0.0394<br>(-0.0421,-0.0368)<br>[p <1e-10]	-0.0380<br>(-0.0426,-0.0334)<br>[p <1e-10]	-0.8273<br>(-0.9176,-0.7369)<br>[p <1e-10]	−−	-0.1786<br>(-0.3572,0.0001)<br>[p=0.0050]	0.1435<br>(-0.0071,0.2941)<br>[p=0.0075]	-0.0843<br>(-0.2121,0.0435)<br>[p=0.064]	0.0160<br>(-0.1480,0.1799)<br>[p=0.78]
Male Daytime SpO_2	0.23	1.10	96.63<br>(96.60,96.66)<br>[p <1e-10]	-0.0282<br>(-0.0297,-0.0267)<br>[p <1e-10]	-0.0509<br>(-0.0542,-0.0476)<br>[p <1e-10]	-0.8427<br>(-0.8932,-0.7922)<br>[p <1e-10]	−−	-0.0897<br>(-0.1688,-0.0106)<br>[p=0.0015]	-0.1506<br>(-0.2444,-0.0568)<br>[p=6.6e-06]	-0.1439<br>(-0.2114,-0.0763)<br>[p=2.3e-09]	-0.0260<br>(-0.1233,0.0713)<br>[p=0.45]
Full Cohort Nocturnal SpO_2	0.32	1.21	95.95<br>(95.91,95.99)<br>[p <1e-10]	-0.0429<br>(-0.0444,-0.0415)<br>[p <1e-10]	-0.0562<br>(-0.0591,-0.0533)<br>[p <1e-10]	-1.1736<br>(-1.2214,-1.1257)<br>[p <1e-10]	-0.0038<br>(-0.0459,0.0383)<br>[p=0.80]	0.0775<br>(-0.0015,0.1564)<br>[p=0.0059]	-0.0068<br>(-0.0930,0.0794)<br>[p=0.82]	0.0243<br>(-0.0406,0.0893)<br>[p=0.29]	0.0596<br>(-0.0311,0.1504)<br>[p=0.065]
Female Nocturnal SpO_2	0.34	1.26	95.93<br>(95.88,95.98)<br>[p <1e-10]	-0.0503<br>(-0.0531,-0.0474)<br>[p <1e-10]	-0.0521<br>(-0.0570,-0.0472)<br>[p <1e-10]	-1.1706<br>(-1.2665,-1.0746)<br>[p <1e-10]	−−	-0.0894<br>(-0.2792,0.1005)<br>[p=0.19]	0.1189<br>(-0.0411,0.2790)<br>[p=0.037]	0.0427<br>(-0.0931,0.1785)<br>[p=0.38]	0.1067<br>(-0.0675,0.2809)<br>[p=0.085]
Male Nocturnal SpO_2	0.32	1.20	95.96<br>(95.92,95.99)<br>[p <1e-10]	-0.0401<br>(-0.0418,-0.0384)<br>[p <1e-10]	-0.0592<br>(-0.0628,-0.0556)<br>[p <1e-10]	-1.1732<br>(-1.2282,-1.1182)<br>[p <1e-10]	−−	0.1115<br>(0.0253,0.1977)<br>[p=2.8e-04]	-0.0558<br>(-0.1580,0.0464)<br>[p=0.13]	0.0235<br>(-0.0501,0.0971)<br>[p=0.37]	0.0416<br>(-0.0644,0.1477)<br>[p=0.27]

Supplementary Table 5: Linear model M_1 fit results for three subject groups (full cohort, female subjects only, and male subjects only), applied to either daytime SpO_2 (top three rows of coefficients) or to nocturnal SpO_2 (bottom three rows of coefficients). Sex variables for the full subject cohort are encoded using a value of 1 for Male subjects and 0 for Female subjects. Race/Ethnicity variables for the full cohort and both sexes encoded using a value of 1 for each listed race/ethnicity group, with White subjects encoded using all zeros for these variables. Values listed in parentheses represent 99.5% confidence intervals for the fitted model coefficients. Values in brackets are regression coefficient p-values, corresponding to two-sided t-tests under the null hypothesis that the coefficient is equal to zero. R^2 = coefficient of determination. SEE = standard error of the estimate.

Linear Regression Results for Blood Oxygen Saturation: Proposed Model $M_{1,race-ethn.}$							
Model	R^2	SEE	Constant	Age<br>(yr)	BMI<br>(kg/m^2)	Altitude<br>(km)	Sex<br>(m=1, f=0)
Asian R/E<br>Daytime SpO_2	0.13	1.16	96.52<br>(96.34,96.69)<br>[p <1e-10]	-0.0216<br>(-0.0277,-0.0155)<br>[p <1e-10]	-0.0498<br>(-0.0646,-0.0349)<br>[p <1e-10]	-0.8041<br>(-1.0358,-0.5724)<br>[p <1e-10]	0.0303<br>(-0.1576,0.2183)<br>[p=0.65]
Black R/E<br>Daytime SpO_2	0.18	1.22	96.81<br>(96.63,96.99)<br>[p <1e-10]	-0.0297<br>(-0.0368,-0.0226)<br>[p <1e-10]	-0.0475<br>(-0.0591,-0.0360)<br>[p <1e-10]	-0.7746<br>(-1.0418,-0.5073)<br>[p <1e-10]	-0.3590<br>(-0.5400,-0.1779)<br>[p=2.9e-08]
Hispanic R/E<br>Daytime SpO_2	0.19	1.17	96.60<br>(96.46,96.73)<br>[p <1e-10]	-0.0297<br>(-0.0350,-0.0244)<br>[p <1e-10]	-0.0498<br>(-0.0587,-0.0409)<br>[p <1e-10]	-0.7169<br>(-0.8588,-0.5751)<br>[p <1e-10]	-0.1485<br>(-0.2855,-0.0116)<br>[p=0.0023]
Other R/E<br>Daytime SpO_2	0.25	1.18	96.72<br>(96.53,96.90)<br>[p <1e-10]	-0.0370<br>(-0.0441,-0.0298)<br>[p <1e-10]	-0.0442<br>(-0.0569,-0.0314)<br>[p <1e-10]	-1.0287<br>(-1.2627,-0.7946)<br>[p <1e-10]	-0.0928<br>(-0.2823,0.0967)<br>[p=0.17]
White R/E<br>Daytime SpO_2	0.25	1.10	96.64<br>(96.60,96.68)<br>[p <1e-10]	-0.0320<br>(-0.0335,-0.0305)<br>[p <1e-10]	-0.0451<br>(-0.0481,-0.0420)<br>[p <1e-10]	-0.8515<br>(-0.9007,-0.8024)<br>[p <1e-10]	-0.0183<br>(-0.0621,0.0254)<br>[p=0.24]
Asian R/E<br>Nocturnal SpO_2	0.21	1.16	95.92<br>(95.74,96.10)<br>[p <1e-10]	-0.0313<br>(-0.0374,-0.0251)<br>[p <1e-10]	-0.0572<br>(-0.0721,-0.0422)<br>[p <1e-10]	-1.1187<br>(-1.3514,-0.8860)<br>[p <1e-10]	0.1530<br>(-0.0357,0.3418)<br>[p=0.023]
Black R/E<br>Nocturnal SpO_2	0.24	1.27	96.02<br>(95.83,96.20)<br>[p <1e-10]	-0.0416<br>(-0.0491,-0.0342)<br>[p <1e-10]	-0.0518<br>(-0.0639,-0.0397)<br>[p <1e-10]	-1.0364<br>(-1.3156,-0.7572)<br>[p <1e-10]	-0.1849<br>(-0.3740,0.0043)<br>[p=0.0061]
Hispanic R/E<br>Nocturnal SpO_2	0.28	1.21	95.96<br>(95.82,96.10)<br>[p <1e-10]	-0.0444<br>(-0.0499,-0.0389)<br>[p <1e-10]	-0.0548<br>(-0.0641,-0.0456)<br>[p <1e-10]	-0.9904<br>(-1.1378,-0.8430)<br>[p <1e-10]	-0.0600<br>(-0.2024,0.0823)<br>[p=0.24]
Other R/E<br>Nocturnal SpO_2	0.35	1.23	96.13<br>(95.93,96.32)<br>[p <1e-10]	-0.0490<br>(-0.0564,-0.0415)<br>[p <1e-10]	-0.0583<br>(-0.0716,-0.0450)<br>[p <1e-10]	-1.3740<br>(-1.6182,-1.1299)<br>[p <1e-10]	-0.0849<br>(-0.2825,0.1128)<br>[p=0.23]
White R/E<br>Nocturnal SpO_2	0.33	1.21	95.95<br>(95.90,96.00)<br>[p <1e-10]	-0.0434<br>(-0.0450,-0.0418)<br>[p <1e-10]	-0.0567<br>(-0.0600,-0.0533)<br>[p <1e-10]	-1.1963<br>(-1.2502,-1.1423)<br>[p <1e-10]	0.0104<br>(-0.0376,0.0585)<br>[p=0.54]

Supplementary Table 6: Linear regression results for participants in each race/ethnicity group, fit to $M_{1,race-ethn.}$ (identical to model M_1, except for the omission of variables encoding race/ethnicity). Models are fit using either daytime SpO_2 as the dependent variable (top five rows of coefficients) or nocturnal SpO_2 as the dependent variable (bottom five rows of coefficients). Sex variables for all models are encoded using a value of 1 for Male subjects and 0 for Female subjects. Values listed in parentheses represent 99.5% confidence intervals for the fitted model coefficients. R^2 = coefficient of determination. SEE = standard error of the estimate.

Supplementary Note 2 (Regression Results for Models Incorporating Linear Interaction Terms for Sex and Race/Ethnicity)

For additional subgroup analysis by sex, model M_1 was modified by adding linear interaction terms based on self-reported sex to yield model $M_{1,sex-interaction}$ described in Supplementary Table 7. Regression results for this model fit to the full subject cohort for $dSpO_2$ and $nSpO_2$ are tabulated in Supplementary Table 8. R_2 and SEE values for $M_{1,sex-interaction}$ are equivalent to those of model M_1, with slightly improved goodness of fit as determined by Bayes Information Criterion (BIC). Comparing the sex interaction regression coefficients from $M_{1,sex-interaction}$ fit to the full subject cohort (rightmost three columns in Supplementary Table 8) with the regression coefficients for $M_{1,sex}$ fit separately to males and females (Supplementary Table 5) shows quantitative agreement between the difference in male-only and female-only $M_{1,sex}$ Age, BMI and altitude regression regression coefficients and the corresponding interaction coefficients of $M_{1,sex-interaction}$ to within a factor of 10^{-6}. Additionally, all statistically significant coefficients for interaction terms in the fitted model match the covariates that were identified via stratified analysis as having a significant sex-dependent difference.

Additional linear regression models incorporating interaction terms for race/ethnicity groups and for combined sex and race/ethnicity groups were investigated, but produced inferior goodness of fit (as determined by BIC) compared to model M_1. Analysis of group differences based on race/ethnicity was accomplished via stratified analysis using M_1 as described in the 'Statistical Analysis' section of the main document.

Model Name	Covariates	Model Usage
$M_{1,sex-interaction}$	Age - 40 (yr)<br>BMI - 25 (kg/m^2)<br>Home altitude (km)<br>Sex(categorical)<br>(Age - 40)*Sex<br>(BMI - 25)*Sex<br>Home altitude*Sex	Sex subgroup analysis.

Supplementary Table 7: Summary of model $M_{1,sex-interaction}$ employed for sex subgroup analysis.

Linear Regression Results for Model $M_{1,sex-interaction}$										
Model	R^2	SEE	Constant	Age (yr)	BMI (kg/m^2)	Altitude (km)	Sex (m=1, f=0)	Age:Male Sex	BMI:Male Sex	Altitude:Male Sex
Full Cohort Daytime SpO_2	0.23	1.12	96.60 (96.56,96.65) [p <1e-10]	-0.0391 (-0.0416,-0.0366) [p <1e-10]	-0.0373 (-0.0416,-0.0329) [p <1e-10]	-0.8275 (-0.9131,-0.7419) [p <1e-10]	-0.0013 (-0.0537,0.0511) [p=0.95]	0.0115 (0.0085,0.0144) [p <1e-10]	-0.0139 (-0.0195,-0.0084) [p <1e-10]	-0.0082 (-0.1081,0.0917) [p=0.82]
Full Cohort Nocturnal SpO_2	0.32	1.21	95.94 (95.89,95.99) [p <1e-10]	-0.0503 (-0.0530,-0.0476) [p <1e-10]	-0.0514 (-0.0561,-0.0467) [p <1e-10]	-1.1720 (-1.2645,-1.0795) [p <1e-10]	0.0284 (-0.0282,0.0851) [p=0.16]	0.0100 (0.0068,0.0132) [p <1e-10]	-0.0083 (-0.0142,-0.0023) [p=9.6e-05]	-0.0048 (-0.1127,0.1031) [p=0.90]

Supplementary Table 8: Linear regression results for Model $M_{1,sex-interaction}$ fit to the full subject cohort using either daytime SpO_2 as the dependent variable (top row of coefficients) or nocturnal SpO_2 as the dependent variable (bottom row of coefficients). Sex interactions are encoded using a value of 1 for Male subjects and 0 for Female subjects. Values listed in parentheses represent 99.5% confidence intervals for the fitted model coefficients. R^2 = coefficient of determination. SEE = standard error of the estimate.

Supplementary Note 3 (SpO_2 Circadian Alignment Using Sleep Tracking Data)

A significant fraction (N=21633, 65%) of study subjects provided nightly sleep tracking data sufficiently overlapping their SpO_2 measurement dates to enable circadian alignment using measured sleep times. For these subjects ('Sleep Cohort'), the timestamp for each SpO_2 measurement was adjusted to represent hours since the most recent start of a reported sleep session (if sleep data was available within the preceding 24 hours). SpO_2 measurements not preceded by a sleep session within the preceding 24 hours were discarded. Using the new measurement timestamps (now corresponding to 00:00 at the start of each night's sleep rather than 00:00 at midnight local clock time), the 24h mean circadian profiles and $dSpO_2$ and $nSpO_2$ were then recalculated for each subject in the Sleep Cohort.

The distribution of all nightly sleep start times relative to midnight clock time are shown in Supplementary Figure 1a. Mean nightly sleep start times for the Sleep Cohort were centered shortly before midnight (median start of sleep 23:35 local clock time, IQR 107 minutes). 24-hour circadian profiles for the original cohort and sleep cohort aligned closely (Supplementary Figure 1b), with $dSpO_2$ and $nSpO_2$ differing between sleep-aligned and original values by mean $\pm$ standard deviation of 0.06 $\pm$ 0.30% and -0.04 $\pm$ 0.27%, respectively. Scatterplots for the original clock-aligned and sleep-aligned $dSpO_2$ and $nSpO_2$ for all subjects in the Sleep Cohort are shown in Supplementary Figures 1c and 1d.

Sleep-aligned $dSpO_2$ and $nSpO_2$ values from the Sleep Cohort were fit using proposed regression model M_1, and the regression coefficients compared against models fit using the original clock-aligned $dSpO_2$ and $nSpO_2$ values from the Sleep Cohort as well as the original clock-aligned values from the full study population. No regression coefficients differed meaningfully for between models fit to the full study population compared to the Sleep Cohort, or for models fit using the sleep cohort using clock-aligned compared to sleep-aligned SpO_2 measurements. A comparison of fitted regression coefficients is shown in Supplementary Figure 2.

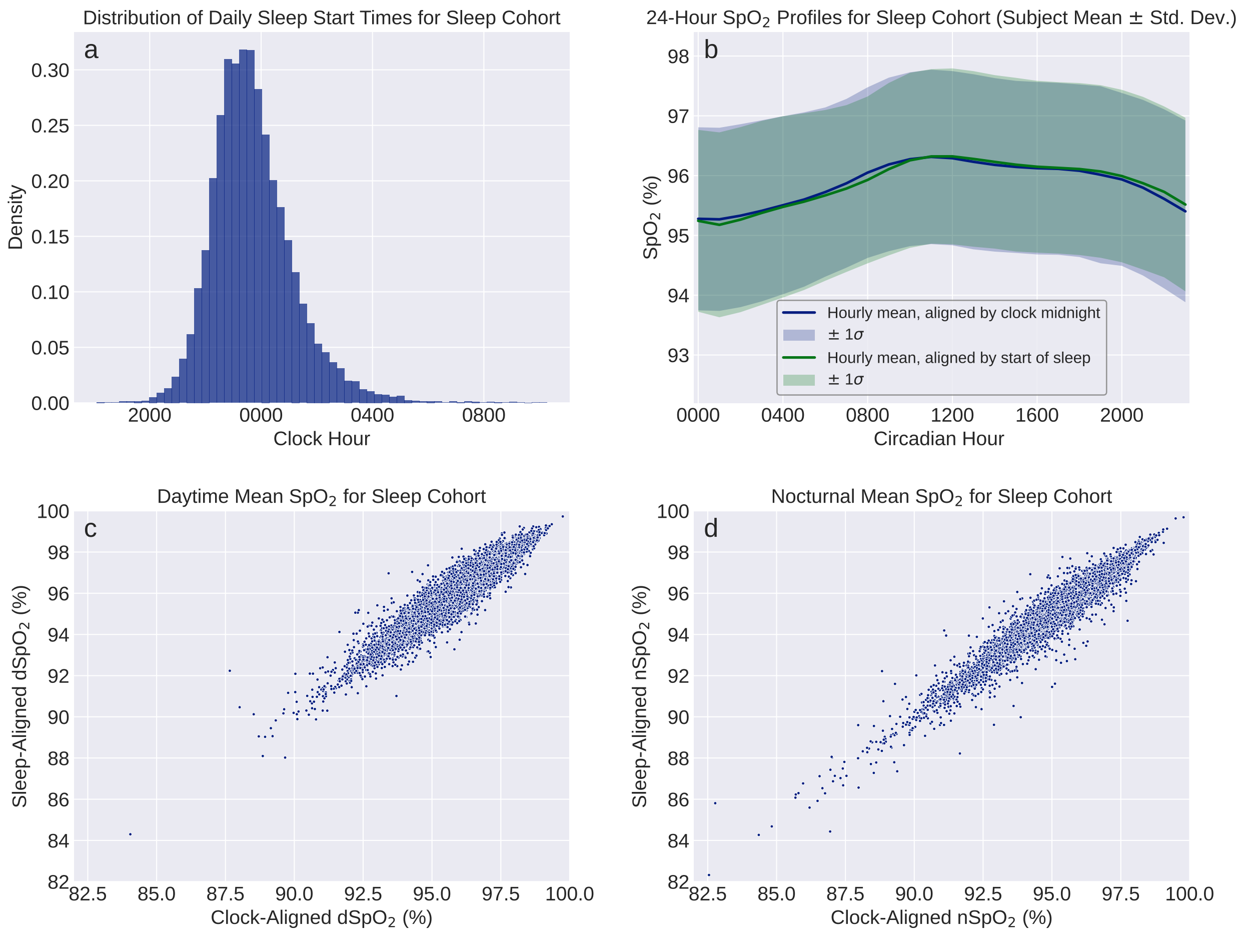

Supplementary Figure 1: Summary of SpO_2 data alignment using sleep tracking information. (a) Distribution of all nightly sleep start times relative to midnight. (b) Comparison of mean SpO_2 24-hour profile based on alignment by clock time with mean SpO_2 24-hour profile based on alignment by sleep start time for all subjects in the Sleep Cohort. (c-d) scatterplots of original clock-aligned and sleep-aligned $dSpO_2$ and $nSpO_2$ for all subjects in theSleep Cohort.

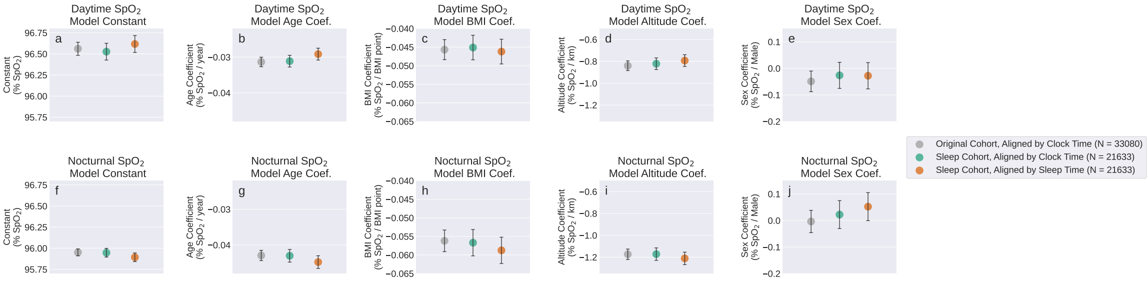

Supplementary Figure 2: Comparison of regression coefficients for M_1 models fit using the full cohort data aligned by clock time (‘Original Cohort, Aligned by Clock Time’), using the Sleep Cohort aligned by clock time (‘Sleep Cohort, Aligned by Clock Time’), and using the Sleep Cohort aligned by sleep tracking measurements (‘Sleep Cohort, Aligned by Sleep Time’), for daytime SpO_2 (a-d, top row) and nocturnal SpO_2 (e-h, bottom row). Error bars represent 99.5% confidence intervals for the fitted coefficients. Race/Ethnicity variables are omitted for clarity. Plotted coefficients and confidence intervals are identical to the values listed in Supplementary Table 9.

Linear Regression Results for Proposed Model (M_1), Comparing Fits Using Data Aligned by Clock Time and Data Aligned by Sleep Start Time											
Model	R^2	SEE	Constant	Age,<br>(yr)	BMI<br>(kg/m^2)	Altitude<br>(km)	Sex<br>(m=1, f=0)	Asian<br>Race/Ethnicity	Black<br>Race/Ethnicity	Hispanic<br>Race/Ethnicity	Other<br>Race/Ethnicity
Original Cohort,<br>Clock Time-Aligned,<br>Daytime SpO_2	0.23	1.12	96.66<br>(96.62,96.70)<br>[p <1e-10]	-0.0314<br>(-0.0327,-0.0300)<br>[p <1e-10]	-0.0457<br>(-0.0484,-0.0430)<br>[p <1e-10]	-0.8402<br>(-0.8845,-0.7959)<br>[p <1e-10]	-0.0490<br>(-0.0880,-0.0101)<br>[p=4.1e-04]	-0.1004<br>(-0.1735,-0.0273)<br>[p=1.2e-04]	-0.0648<br>(-0.1446,0.0150)<br>[p=0.023]	-0.1334<br>(-0.1936,-0.0733)<br>[p=4.8e-10]	-0.0146<br>(-0.0986,0.0694)<br>[p=0.63]
Sleep Cohort,<br>Clock Time-Aligned,<br>Daytime SpO_2	0.23	1.11	96.66<br>(96.61,96.71)<br>[p <1e-10]	-0.0311<br>(-0.0328,-0.0295)<br>[p <1e-10]	-0.0451<br>(-0.0484,-0.0418)<br>[p <1e-10]	-0.8217<br>(-0.8749,-0.7685)<br>[p <1e-10]	-0.0263<br>(-0.0755,0.0228)<br>[p=0.13]	-0.1330<br>(-0.2265,-0.0395)<br>[p=6.5e-05]	-0.0307<br>(-0.1392,0.0777)<br>[p=0.43]	-0.1701<br>(-0.2537,-0.0866)<br>[p=1.1e-08]	0.1040<br>(-0.0383,0.2462)<br>[p=0.040]
Sleep Cohort,<br>Sleep Time-Aligned,<br>Daytime SpO_2	0.22	1.12	96.76<br>(96.71,96.81)<br>[p <1e-10]	-0.0292<br>(-0.0308,-0.0275)<br>[p <1e-10]	-0.0462<br>(-0.0495,-0.0429)<br>[p <1e-10]	-0.7932<br>(-0.8467,-0.7396)<br>[p <1e-10]	-0.0279<br>(-0.0774,0.0216)<br>[p=0.11]	-0.1458<br>(-0.2399,-0.0517)<br>[p=1.4e-05]	-0.0786<br>(-0.1878,0.0305)<br>[p=0.043]	-0.1558<br>(-0.2399,-0.0718)<br>[p=2.0e-07]	0.0776<br>(-0.0656,0.2209)<br>[p=0.13]
Original Cohort,<br>Clock Time-Aligned,<br>Nocturnal SpO_2	0.32	1.21	95.95<br>(95.91,95.99)<br>[p <1e-10]	-0.0429<br>(-0.0444,-0.0415)<br>[p <1e-10]	-0.0562<br>(-0.0591,-0.0533)<br>[p <1e-10]	-1.1736<br>(-1.2214,-1.1257)<br>[p <1e-10]	-0.0038<br>(-0.0459,0.0383)<br>[p=0.80]	0.0775<br>(-0.0015,0.1564)<br>[p=0.0059]	-0.0068<br>(-0.0930,0.0794)<br>[p=0.82]	0.0243<br>(-0.0406,0.0893)<br>[p=0.29]	0.0596<br>(-0.0311,0.1504)<br>[p=0.065]
Sleep Cohort,<br>Clock Time-Aligned,<br>Nocturnal SpO_2	0.33	1.19	95.95<br>(95.90,96.00)<br>[p <1e-10]	-0.0430<br>(-0.0448,-0.0413)<br>[p <1e-10]	-0.0567<br>(-0.0602,-0.0531)<br>[p <1e-10]	-1.1722<br>(-1.2293,-1.1151)<br>[p <1e-10]	0.0223<br>(-0.0304,0.0750)<br>[p=0.24]	0.0392<br>(-0.0611,0.1396)<br>[p=0.27]	0.0288<br>(-0.0875,0.1452)<br>[p=0.49]	0.0144<br>(-0.0752,0.1040)<br>[p=0.65]	0.0572<br>(-0.0955,0.2098)<br>[p=0.29]
Sleep Cohort,<br>Sleep Time-Aligned,<br>Nocturnal SpO_2	0.35	1.19	95.89<br>(95.84,95.94)<br>[p <1e-10]	-0.0448<br>(-0.0466,-0.0430)<br>[p <1e-10]	-0.0588<br>(-0.0623,-0.0552)<br>[p <1e-10]	-1.2112<br>(-1.2683,-1.1541)<br>[p <1e-10]	0.0521<br>(-0.0006,0.1049)<br>[p=0.0056]	0.1094<br>(0.0090,0.2098)<br>[p=0.0022]	0.0616<br>(-0.0548,0.1781)<br>[p=0.14]	0.0683<br>(-0.0214,0.1580)<br>[p=0.033]	0.0713<br>(-0.0814,0.2241)<br>[p=0.19]

Supplementary Table 9: Linear regression results for M_1 models fit to the original full cohort aligned by clock time, fit to the Sleep Cohort for SpO_2 aligned by clock time, and fit to the Sleep Cohort aligned by sleep start time. Models are fit using either daytime SpO_2 as the dependent variable (top 3 rows of coefficients) or nocturnal SpO_2 as the dependent variable (bottom 3 rows of coefficients). Sex variables for all subgroups are encoded using a value of 1 for Male subjects and 0 for Female subjects. Values listed in parentheses represent 99.5% confidence intervals for the fitted model coefficients. Values in brackets are regression coefficient p-values, corresponding to two-sided t-tests under the null hypothesis that the coefficient is equal to zero. R^2 = coefficient of determination. SEE = standard error of the estimate.

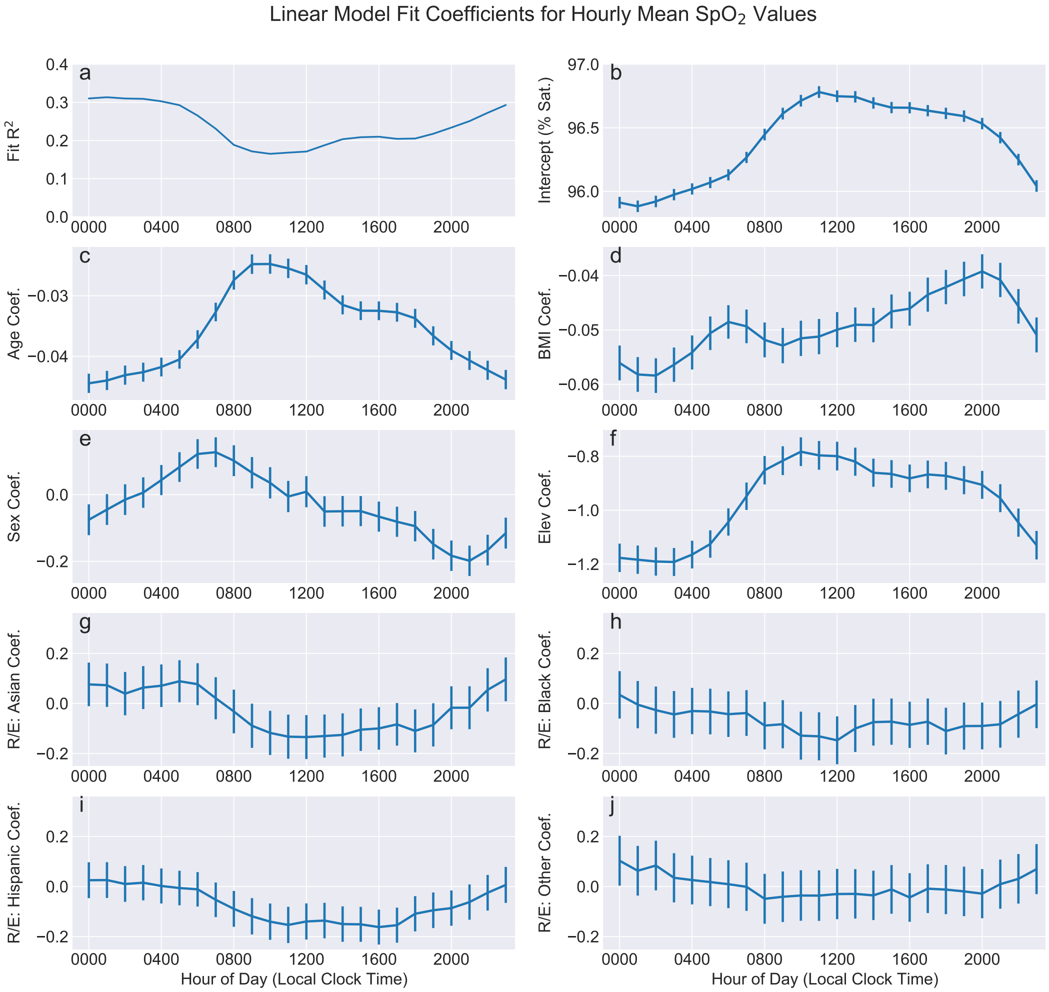

Supplementary Figure 3: Linear regression R^2 and model coefficients produced by fitting M_1 using subject-mean SpO_2 aggregated for each individual hour of the day: (a) Fitted R^2 is highest during typical sleep hours (approx. 23:00–06:00) compared with daytime hours. (b) Intercept, (c) age, (d) BMI, and (f) altitude coefficients exhibit clear circadian variation and have greatest absolute magnitude during typical sleep hours. Regression coefficients for (e) assigned sex and (g-j) race/ethnicity group also exhibit a small degree of diurnal variation. In all plots, error whiskers correspond to 99.5% confidence intervals. R^2 = coefficient of determination.

Supplementary Note 4 (Influence of Reduced Data Timespan on Regression Results)

As described in the Methods section of the primary document, $dSpO_2$ and $nSpO_2$ values for each subject used in the regression modeling analysis were calculated by averaging all daytime and nocturnal measurements captured over the full timespan of the data set (up to 37 weeks per subject). To evaluate the impact of reducing the data timespan, we separately calculated $dSpO_2$ and $nSpO_2$ while limiting the total data timespan to a maximum of 30 calendar days for each subject. Many subjects in the data set did not produce SpO_2 on every calendar day, so this necessitated the use of a rolling 30-day date window for each subject, with $dSpO_2$ and $nSpO_2$ calculated from the most recent 30-day date window in which each subject satisfied the data coverage requirements described in the Methods section. A total of 29,556 subjects ('Rolling 30d Cohort') satisfied this requirement, with the remaining 3,524 subjects failing to meet daytime and nocturnal measurement count requirements within any 30-day date window.

Model M_1 was fit using $dSpO_2$ and $nSpO_2$ values for the Rolling 30d Cohort calculated from 30 days of data per subject, as well as for the same cohort using the full study timespan. Regression coefficients for the fitted models are compared with those of the full subject cohort in Supplementary Figure 4, and tabulated in Supplementary Table 10. No pairwise comparisons between regression coefficients indicated statistically significant differences between the full study period and the 30-day time-windowed data.

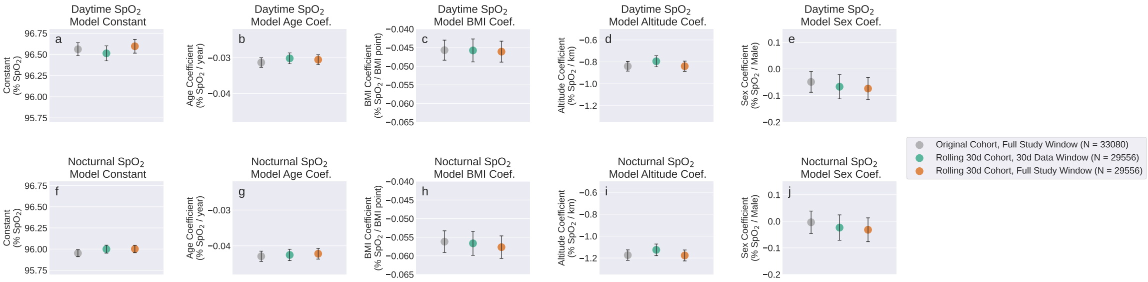

Supplementary Figure 4: Comparison of regression coefficients for M_1 models fit to the full cohort using SpO_2 measurements from all available dates for each subject ('Original Cohort, Full Study Window'), for a subset of subjects with SpO_2 measurements limited to a maximum timespan of 30 calendar days ('Rolling 30d Cohort, 30d Data Window'), and for the same subset of subjects using SpO_2 measurements from all available dates ('Rolling 30d Cohort, Full Study Window'), for daytime SpO_2 (a-d, top row) and nocturnal SpO_2 (e-h, bottom row). Error bars represent 99.5% confidence intervals for the fitted coefficients. Race/Ethnicity variables are omitted for clarity. Plotted coefficients and confidence intervals are identical to the values listed in Supplementary Table 10.

Linear Regression Results for Proposed Model (M_1), Comparing Fits Using Data from Full Study Duration with Fits Using Data from 30-Day Limited Date Range per Subject

Model	R^2	SEE	Constant	Age (yr)	BMI (kg/m^2)	Altitude (km)	Sex (m=1, f=0)	Asian Race/Ethnicity	Black Race/Ethnicity	Hispanic Race/Ethnicity	Other Race/Ethnicity
Original Cohort,<br>Full Study Window<br>Daytime SpO_2	0.23	1.12	96.66<br>(96.62,96.70)<br>[p <1e-10]	-0.0314<br>(-0.0327,-0.0300)<br>[p <1e-10]	-0.0457<br>(-0.0484,-0.0430)<br>[p <1e-10]	-0.8402<br>(-0.8845,-0.7959)<br>[p <1e-10]	-0.0490<br>(-0.0880,-0.0101)<br>[p=4.1e-04]	-0.1004<br>(-0.1735,-0.0273)<br>[p=1.2e-04]	-0.0648<br>(-0.1446,0.0150)<br>[p=0.023]	-0.1334<br>(-0.1936,-0.0733)<br>[p=4.8e-10]	-0.0146<br>(-0.0986,0.0694)<br>[p=0.63]
Rolling 30d Cohort,<br>30d Data Window<br>Daytime SpO_2	0.19	1.22	96.65<br>(96.60,96.69)<br>[p <1e-10]	-0.0302<br>(-0.0317,-0.0287)<br>[p <1e-10]	-0.0458<br>(-0.0488,-0.0427)<br>[p <1e-10]	-0.7948<br>(-0.8456,-0.7439)<br>[p <1e-10]	-0.0671<br>(-0.1127,-0.0215)<br>[p=3.6e-05]	-0.1348<br>(-0.2181,-0.0515)<br>[p=5.6e-06]	-0.0643<br>(-0.1558,0.0273)<br>[p=0.049]	-0.1626<br>(-0.2317,-0.0936)<br>[p <1e-10]	-0.0188<br>(-0.1154,0.0778)<br>[p=0.59]
Rolling 30d Cohort,<br>Full Study Window<br>Daytime SpO_2	0.23	1.11	96.70<br>(96.66,96.74)<br>[p <1e-10]	-0.0306<br>(-0.0320,-0.0292)<br>[p <1e-10]	-0.0461<br>(-0.0489,-0.0433)<br>[p <1e-10]	-0.8399<br>(-0.8863,-0.7935)<br>[p <1e-10]	-0.0740<br>(-0.1156,-0.0324)<br>[p=6.1e-07]	-0.1030<br>(-0.1791,-0.0269)<br>[p=1.4e-04]	-0.0626<br>(-0.1463,0.0210)<br>[p=0.035]	-0.1426<br>(-0.2056,-0.0795)<br>[p=2.2e-10]	0.0044<br>(-0.0838,0.0927)<br>[p=0.89]
Original Cohort,<br>Full Study Window<br>Nocturnal SpO_2	0.32	1.21	95.95<br>(95.91,95.99)<br>[p <1e-10]	-0.0429<br>(-0.0444,-0.0415)<br>[p <1e-10]	-0.0562<br>(-0.0591,-0.0533)<br>[p <1e-10]	-1.1736<br>(-1.2214,-1.1257)<br>[p <1e-10]	-0.0038<br>(-0.0459,0.0383)<br>[p=0.80]	0.0775<br>(-0.0015,0.1564)<br>[p=0.0059]	-0.0068<br>(-0.0930,0.0794)<br>[p=0.82]	0.0243<br>(-0.0406,0.0893)<br>[p=0.29]	0.0596<br>(-0.0311,0.1504)<br>[p=0.065]
Rolling 30d Cohort,<br>30d Data Window<br>Nocturnal SpO_2	0.30	1.28	96.00<br>(95.95,96.05)<br>[p <1e-10]	-0.0426<br>(-0.0442,-0.0410)<br>[p <1e-10]	-0.0566<br>(-0.0599,-0.0534)<br>[p <1e-10]	-1.1255<br>(-1.1788,-1.0723)<br>[p <1e-10]	-0.0238<br>(-0.0715,0.0240)<br>[p=0.16]	0.0642<br>(-0.0231,0.1515)<br>[p=0.039]	-0.0098<br>(-0.1057,0.0862)<br>[p=0.78]	0.0065<br>(-0.0658,0.0789)<br>[p=0.80]	0.0586<br>(-0.0426,0.1598)<br>[p=0.10]
Rolling 30d Cohort,<br>Full Study Window<br>Nocturnal SpO_2	0.33	1.20	96.00<br>(95.96,96.05)<br>[p <1e-10]	-0.0422<br>(-0.0437,-0.0407)<br>[p <1e-10]	-0.0577<br>(-0.0607,-0.0546)<br>[p <1e-10]	-1.1766<br>(-1.2265,-1.1266)<br>[p <1e-10]	-0.0320<br>(-0.0768,0.0128)<br>[p=0.045]	0.0698<br>(-0.0121,0.1518)<br>[p=0.017]	-0.0088<br>(-0.0988,0.0812)<br>[p=0.78]	0.0188<br>(-0.0491,0.0867)<br>[p=0.44]	0.0762<br>(-0.0188,0.1711)<br>[p=0.024]

Supplementary Table 10: Linear regression results for M_1 models fit to the full cohort using SpO_2 measurements from all available dates for each subject ('Original Cohort, Full Study Window'), for a subset of subjects with SpO_2 measurements limited to a maximum timespan of 30 calendar days ('Rolling 30d Cohort, 30d Data Window'), and for the same subset of subjects using SpO_2 measurements from all available dates ('Rolling 30d Cohort, Full Study Window'). Models are fit using either daytime SpO_2 as the dependent variable (top 3 rows of coefficients) or nocturnal SpO_2 as the dependent variable (bottom 3 rows of coefficients). Sex variables for all subgroups are encoded using a value of 1 for Male subjects and 0 for Female subjects. Values listed in parentheses represent 99.5% confidence intervals for the fitted model coefficients. Values in brackets are regression coefficient p-values, corresponding to two-sided t-tests under the null hypothesis that the coefficient is equal to zero. R^2 = coefficient of determination. SEE = standard error of the estimate.

Supplementary Note 5 (Influence of Chronic Health Conditions and Smoking Habits on Regression Results)

To analyze whether the systematic SpO_2 trends measured with respect to Age and BMI may arise due to accumulation of chronic lung disease or use of cigarettes, we fit model M_1 after grouping subjects based on self report of chronic lung disease and cigarette use. From the full cohort of 33,080 subjects, 980 subjects (3.0%) were assigned to a 'Current Smokers' cohort based on self-report of current daily cigarette use (irrespective of self-reported health conditions). 1,015 subjects (3.1%) were assigned to an 'Unhealthy' cohort based on self-report of chronic bronchitis, chronic obstructive pulmonary disease, emphysema, or any form of heart failure. 21,631 subjects (65.4%) were assigned a 'Healthy Lifetime Nonsmokers' cohort based on self-report of no historic or current cigarette use and no self-report of chronic bronchitis, chronic obstructive pulmonary disease, emphysema, or any form of heart failure.

Model M_1 was fit using $dSpO_2$ and $nSpO_2$ values for each health condition/smoking history cohort. Regression coefficients for the fitted models are compared with those of the full subject cohort in Supplementary Figure 5, and tabulated in Supplementary Table 11. Current smokers and subjects with cardiopulmonary disease exhibit significantly lower $nSpO_2$ intercept terms and significantly greater decline in both $dSpO_2$ and $nSpO_2$ with Age, compared with healthy lifetime nonsmokers. Comparison of M_1 fit coefficients from the full cohort with fit coefficients from healthy lifetime nonsmokers shows equivalent decline in both SpO_2 with increasing Age and BMI both groups.

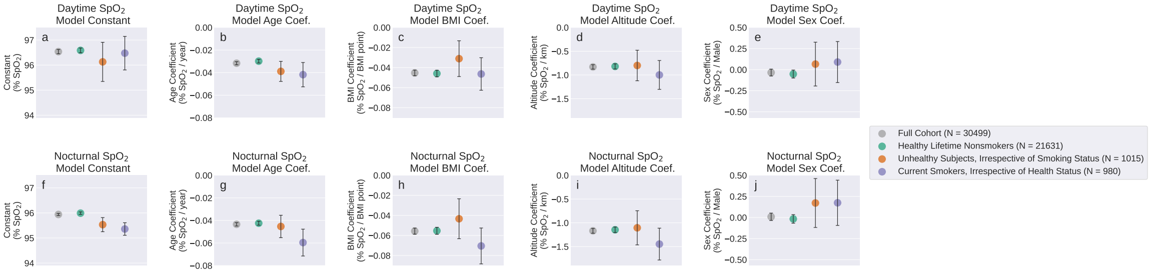

Supplementary Figure 5: Comparison of regression coefficients for M_1 models fit for subjects stratified by self-reported health conditions and smoking habits, for daytime SpO_2 (a-d, top row) and nocturnal SpO_2 (e-h, bottom row). Error bars represent 99.5% confidence intervals for the fitted coefficients. Race/Ethnicity variables are omitted for clarity. Plotted coefficients and confidence intervals are identical to the values listed in Supplementary Table 11.

Linear Regression Results for Proposed Model (M_1), Comparing Fits Using Data from Different Subject Groups Based on Self-Reported Heath Conditions and Smoking Behavior											
Model	R^2	SEE	Constant	Age<br>(yr)	BMI<br>(kg/m^2)	Altitude<br>(km)	Sex<br>(m=1, f=0)	Asian<br>Race/Ethnicity	Black<br>Race/Ethnicity	Hispanic<br>Race/Ethnicity	Other<br>Race/Ethnicity
Full Cohort<br>Daytime SpO_2	0.23	1.12	96.66<br>(96.62,96.70)<br>[p <1e-10]	-0.0314<br>(-0.0327,-0.0300)<br>[p <1e-10]	-0.0457<br>(-0.0484,-0.0430)<br>[p <1e-10]	-0.8402<br>(-0.8845,-0.7959)<br>[p <1e-10]	-0.0490<br>(-0.0880,-0.0101)<br>[p=4.1e-04]	-0.1004<br>(-0.1735,-0.0273)<br>[p=1.2e-04]	-0.0648<br>(-0.1446,0.0150)<br>[p=0.023]	-0.1334<br>(-0.1936,-0.0733)<br>[p=4.8e-10]	-0.0146<br>(-0.0986,0.0694)<br>[p=0.63]
Healthy Lifetime Nonsmokers<br>Daytime SpO_2	0.23	1.10	96.69<br>(96.65,96.74)<br>[p <1e-10]	-0.0299<br>(-0.0315,-0.0282)<br>[p <1e-10]	-0.0459<br>(-0.0492,-0.0426)<br>[p <1e-10]	-0.8197<br>(-0.8735,-0.7658)<br>[p <1e-10]	-0.0520<br>(-0.0995,-0.0044)<br>[p=0.0021]	-0.1078<br>(-0.1962,-0.0195)<br>[p=6.1e-04]	-0.0576<br>(-0.1560,0.0408)<br>[p=0.10]	-0.1848<br>(-0.2640,-0.1056)<br>[p <1e-10]	0.1474<br>(0.0028,0.2919)<br>[p=0.0042]
Unhealthy Subjects<br>Daytime SpO_2	0.19	1.42	96.30<br>(96.04,96.55)<br>[p <1e-10]	-0.0389<br>(-0.0478,-0.0300)<br>[p <1e-10]	-0.0311<br>(-0.0489,-0.0133)<br>[p=1.1e-06]	-0.8003<br>(-1.1219,-0.4788)<br>[p <1e-10]	0.0659<br>(-0.1939,0.3256)<br>[p=0.48]	-0.1676<br>(-0.9304,0.5951)<br>[p=0.54]	-0.1639<br>(-0.7470,0.4192)<br>[p=0.43]	-0.0057<br>(-0.5066,0.4951)<br>[p=0.97]	-0.0650<br>(-0.7052,0.5752)<br>[p=0.78]
Current Smokers<br>Daytime SpO_2	0.23	1.27	96.06<br>(95.83,96.28)<br>[p <1e-10]	-0.0418<br>(-0.0526,-0.0310)<br>[p <1e-10]	-0.0464<br>(-0.0626,-0.0302)<br>[p <1e-10]	-0.9988<br>(-1.3023,-0.6953)<br>[p <1e-10]	0.0902<br>(-0.1531,0.3334)<br>[p=0.30]	0.4188<br>(-0.2321,1.0697)<br>[p=0.071]	0.0914<br>(-0.3985,0.5813)<br>[p=0.60]	0.1498<br>(-0.3548,0.6543)<br>[p=0.40]	-0.0686<br>(-0.6047,0.4676)<br>[p=0.72]
Full Cohort<br>Nocturnal SpO_2	0.32	1.21	95.95<br>(95.91,95.99)<br>[p <1e-10]	-0.0429<br>(-0.0444,-0.0415)<br>[p <1e-10]	-0.0562<br>(-0.0591,-0.0533)<br>[p <1e-10]	-1.1736<br>(-1.2214,-1.1257)<br>[p <1e-10]	-0.0038<br>(-0.0459,0.0383)<br>[p=0.80]	0.0775<br>(-0.0015,0.1564)<br>[p=0.0059]	-0.0068<br>(-0.0930,0.0794)<br>[p=0.82]	0.0243<br>(-0.0406,0.0893)<br>[p=0.29]	0.0596<br>(-0.0311,0.1504)<br>[p=0.065]
Healthy Lifetime Nonsmokers<br>Nocturnal SpO_2	0.33	1.18	95.99<br>(95.94,96.04)<br>[p <1e-10]	-0.0425<br>(-0.0442,-0.0407)<br>[p <1e-10]	-0.0553<br>(-0.0588,-0.0518)<br>[p <1e-10]	-1.1468<br>(-1.2045,-1.0890)<br>[p <1e-10]	-0.0170<br>(-0.0680,0.0339)<br>[p=0.35]	0.0505<br>(-0.0443,0.1452)<br>[p=0.13]	-0.0061<br>(-0.1116,0.0994)<br>[p=0.87]	0.0043<br>(-0.0806,0.0892)<br>[p=0.89]	0.0723<br>(-0.0827,0.2273)<br>[p=0.19]
Unhealthy Subjects<br>Nocturnal SpO_2	0.23	1.58	95.53<br>(95.25,95.81)<br>[p <1e-10]	-0.0453<br>(-0.0553,-0.0354)<br>[p <1e-10]	-0.0433<br>(-0.0632,-0.0234)<br>[p=1.4e-09]	-1.1052<br>(-1.4644,-0.7460)<br>[p <1e-10]	0.1717<br>(-0.1185,0.4619)<br>[p=0.096]	0.0933<br>(-0.7587,0.9454)<br>[p=0.76]	-0.0674<br>(-0.7189,0.5840)<br>[p=0.77]	0.1789<br>(-0.3807,0.7384)<br>[p=0.37]	-0.0294<br>(-0.7446,0.6858)<br>[p=0.91]
Current Smokers<br>Nocturnal SpO_2	0.34	1.41	95.36<br>(95.11,95.61)<br>[p <1e-10]	-0.0596<br>(-0.0715,-0.0477)<br>[p <1e-10]	-0.0704<br>(-0.0883,-0.0525)<br>[p <1e-10]	-1.4477<br>(-1.7826,-1.1127)<br>[p <1e-10]	0.1742<br>(-0.0943,0.4427)<br>[p=0.068]	0.3270<br>(-0.3915,1.0454)<br>[p=0.20]	0.2168<br>(-0.3238,0.7575)<br>[p=0.26]	0.4188<br>(-0.1381,0.9757)<br>[p=0.035]	-0.0425<br>(-0.6342,0.5493)<br>[p=0.84]

Supplementary Table 11: Linear regression results for M_1 models fit for subjects stratified by self-reported health conditions and smoking habits. Models are fit using either daytime SpO_2 as the dependent variable (top 4 rows of coefficients) or nocturnal SpO_2 as the dependent variable (bottom 4 rows of coefficients). Sex variables for all subgroups are encoded using a value of 1 for Male subjects and 0 for Female subjects. Values listed in parentheses represent 99.5% confidence intervals for the fitted model coefficients. Values in brackets are regression coefficient p-values, corresponding to two-sided t-tests under the null hypothesis that the coefficient is equal to zero. R^2 = coefficient of determination. SEE = standard error of the estimate.

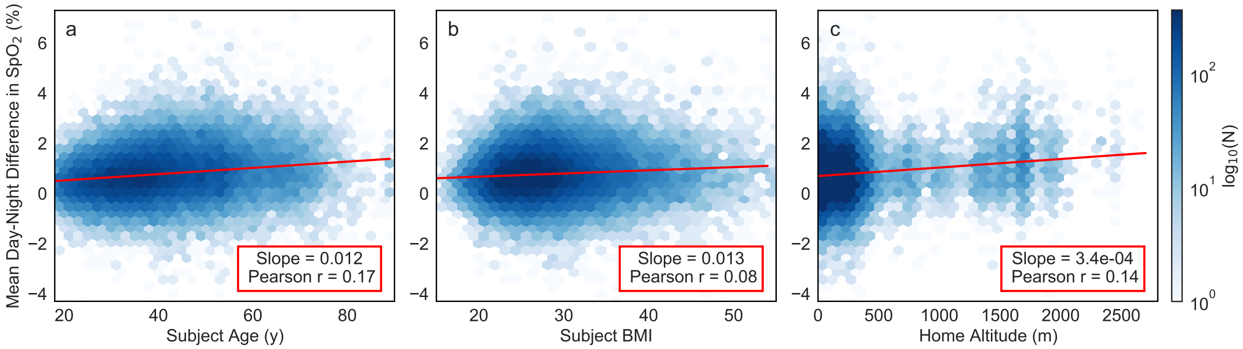

Supplementary Figure 6: Linear relationships between mean day-night SpO_2 difference (dnΔSpO_2) and the three independent variables exhibiting the strongest correlation with these metrics: (a) age, (b) BMI, (c) home altitude. Each plot presents a 2-dimensional histogram of values from all 33,080 subjects in evenly-spaced hexagonal bins, with the color density corresponding to log-scaled bin counts for clarity. Positive values for dnΔSpO_2 correspond to an overnight drop in measured SpO_2. In each plot, the overlaid red line represents the simple univariate linear regression fit using the independent variable shown on the x-axis. The listed slope and Pearson correlation coefficient correspond to the same univariate linear fit.

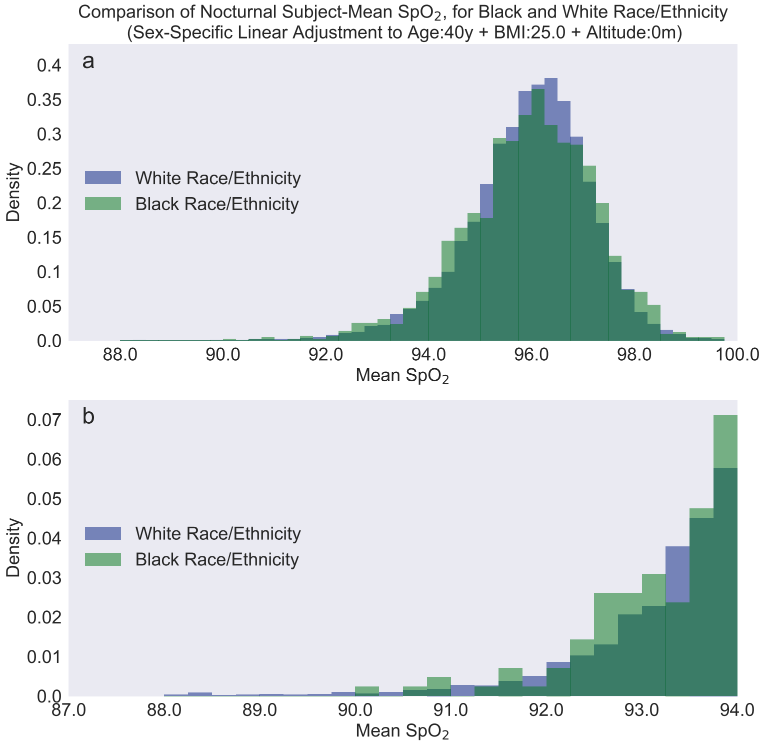

Supplementary Figure 7: Histograms of nocturnal SpO_2 for Black and White subjects, after linear adjustment of age to a target of 40 years, BMI to a target of 25.0 kg/m^2, and home altitude to zero elevation. Distributions are shown for the full range of nocturnal SpO_2 (a) and for the low-saturation range (b). Data for both plots is identical, with the only the axes limits differing. The distributions do not differ with statistical significance ($p > .05$) based on two-sample Kolmogorov-Smirnov test using two-sided alternative hypothesis, either over the full range of SpO_2 or if the distributions are clipped at 94% saturation to emphasize the hypoxic tail.
