# Supplementary material for: Pulse oximetry values from 33,080 participants in the Apple Heart & Movement Study
Source: NPJ Digit Med. 2023 Jul 27;6:134. doi: 10.1038/s41746-023-00851-6 (PMC10374661; doi:10.1038/s41746-023-00851-6)
Supplement: Supplementary file 2 — Reporting Summary [file 41746_2023_851_MOESM2_ESM.pdf]

nature portfolio

Corresponding author(s): Michael O'Reilly

Last updated by author(s): Nov 10, 2022

Reporting Summary

Nature Portfolio wishes to improve the reproducibility of the work that we publish. This form provides structure for consistency and transparency in reporting. For further information on Nature Portfolio policies, see our Editorial Policies and the Editorial Policy Checklist.

Statistics

For all statistical analyses, confirm that the following items are present in the figure legend, table legend, main text, or Methods section.

n/a	Confirmed	
☐	☒	The exact sample size (*n*) for each experimental group/condition, given as a discrete number and unit of measurement
☐	☒	A statement on whether measurements were taken from distinct samples or whether the same sample was measured repeatedly
☐	☒	The statistical test(s) used AND whether they are one- or two-sided<br>*Only common tests should be described solely by name; describe more complex techniques in the Methods section.*
☐	☒	A description of all covariates tested
☐	☒	A description of any assumptions or corrections, such as tests of normality and adjustment for multiple comparisons
☐	☒	A full description of the statistical parameters including central tendency (e.g. means) or other basic estimates (e.g. regression coefficient) AND variation (e.g. standard deviation) or associated estimates of uncertainty (e.g. confidence intervals)
☐	☒	For null hypothesis testing, the test statistic (e.g. *F*, *t*, *r*) with confidence intervals, effect sizes, degrees of freedom and *P* value noted<br>*Give P values as exact values whenever suitable.*
☒	☐	For Bayesian analysis, information on the choice of priors and Markov chain Monte Carlo settings
☒	☐	For hierarchical and complex designs, identification of the appropriate level for tests and full reporting of outcomes
☐	☒	Estimates of effect sizes (e.g. Cohen's *d*, Pearson's *r*), indicating how they were calculated

Our web collection on statistics for biologists contains articles on many of the points above.

Software and code

Policy information about availability of computer code

Data collection	All data collection, both raw measurements and metadata, was accomplished using the using the Apple Research app (versions 2.0.3 through 2.2)
Data analysis	All data analysis code was written in Python 3.6. Plotting and data visualization were performed using the Python packages Seaborn (version 0.11.0) and Matplotlib (version 3.2.2). Ordinary least squares linear regression modeling (OLS) and variance components analysis (VCA) was performed using the Python statsmodels package (version 0.11.1).

For manuscripts utilizing custom algorithms or software that are central to the research but not yet described in published literature, software must be made available to editors and reviewers. We strongly encourage code deposition in a community repository (e.g. GitHub). See the Nature Portfolio guidelines for submitting code & software for further information.

Data

Policy information about availability of data

All manuscripts must include a data availability statement. This statement should provide the following information, where applicable:

- Accession codes, unique identifiers, or web links for publicly available datasets
- A description of any restrictions on data availability
- For clinical datasets or third party data, please ensure that the statement adheres to our policy

The aggregated data that support the findings of this study can be made available on request from the corresponding author (M.O.). Request for data will be evaluated and responded to in a manner consistent with the specific language in the study protocol and informed consent form.

Human research participants

Policy information about studies involving human research participants and Sex and Gender in Research.

Reporting on sex and gender	All analysis and reporting applies to Sex (biological sex assigned at birth). Sex was used in study design. Sex was determined by participant self-report. Sex-stratified analysis was performed and reported. The study cohort consisted of 23911 Male subjects and 9169 Female subjects.
Population characteristics	Total study cohort 33,080 subjects. Mean age 41.03 years. Mean BMI 28.88. Subject counts according to self-reported Race/ethnicity: asian 2063, black 1687, hispanic 3162, other 1501, white 24667.
Recruitment	Subjects were recruited via self-enrollment in the study using the Apple Research app. All participants were users of personally-owned Apple iPhone and Watch devices. Ownership of these products suggests participants are likely to be of higher socioeconomic means compared to US population average. These biases are not expected to influence reported results.
Ethics oversight	Advarra Central Institutional Review Board

Note that full information on the approval of the study protocol must also be provided in the manuscript.

Field-specific reporting

Please select the one below that is the best fit for your research. If you are not sure, read the appropriate sections before making your selection.

☒ Life sciences ☐ Behavioural & social sciences ☐ Ecological, evolutionary & environmental sciences

For a reference copy of the document with all sections, see nature.com/documents/nr-reporting-summary-flat.pdf

Life sciences study design

All studies must disclose on these points even when the disclosure is negative.

Sample size	Sample size (33,080 participants) was based on all available subjects satisfying data coverage requirements. This sample size is expected to be of sufficient size for statistical conclusions based on the low complexity of the linear regression models employed for analysis (maximum 9 fitted regression parameters)
Data exclusions	All data was collected from Jan 1 2021 through Sept 15 2021. All subjects satisfying the data coverage requirements (described in Methods and illustrated in the subject inclusion flowchart) were used in the analysis. No data points were removed from the selected subjects.
Replication	Statistical conclusions were replicated by analysis of time-windowed subsets of the data.
Randomization	This study was cross-sectional in nature. No interventions were performed.
Blinding	This study was cross-sectional in nature. No blinding was appropriate.

Reporting for specific materials, systems and methods

We require information from authors about some types of materials, experimental systems and methods used in many studies. Here, indicate whether each material, system or method listed is relevant to your study. If you are not sure if a list item applies to your research, read the appropriate section before selecting a response.

Materials & experimental systems

n/a	Involved in the study
☒	☐ Antibodies
☒	☐ Eukaryotic cell lines
☒	☐ Palaeontology and archaeology
☒	☐ Animals and other organisms
☐	☒ Clinical data
☒	☐ Dual use research of concern

Methods

n/a	Involved in the study
☒	☐ ChIP-seq
☒	☐ Flow cytometry
☒	☐ MRI-based neuroimaging

Clinical data

Policy information about clinical studies
All manuscripts should comply with the ICMJE guidelines for publication of clinical research and a completed CONSORT checklist must be included with all submissions.

Clinical trial registration	NCT04198194
Study protocol	The study protocol/methods will be described in a forthcoming publication authored by the study PI.
Data collection	Participants self-enrolled in the study. All data collection was accomplished by Apple Watch devices utilizing the Apple Research app.
Outcomes	Outcomes consisted of measured mean daytime and nocturnal SpO2 values. These measures were directly captured by the data collection device (Apple Watch SpO2 sensor)
